# HIV-specific T cell responses reflect substantive in vivo interactions with antigen despite long-term therapy

**DOI:** 10.1172/jci.insight.142640

**Published:** 2021-02-08

**Authors:** Eva M. Stevenson, Adam R. Ward, Ronald Truong, Allison S. Thomas, Szu-Han Huang, Thomas R. Dilling, Sandra Terry, John K. Bui, Talia M. Mota, Ali Danesh, Guinevere Q. Lee, Andrea Gramatica, Pragya Khadka, Winiffer D. Conce Alberto, Rajesh T. Gandhi, Deborah K. McMahon, Christina M. Lalama, Ronald J. Bosch, Bernard Macatangay, Joshua C. Cyktor, Joseph J. Eron, John W. Mellors, R. Brad Jones, for the AIDS Clinical Trials Group A5321 Team

**Affiliations:** 1 Division of Infectious Diseases, Department of Medicine, Weill Cornell Medicine, New York, New York, USA.; 2 Department of Microbiology, Immunology, and Tropical Medicine, School of Medicine & Health Sciences, and; 3 PhD Program in Epidemiology, Department of Epidemiology, Milken Institute School of Public Health, George Washington University, Washington, DC, USA.; 4 Department of Microbiology, Boston University School of Medicine, Boston, Massachusetts, USA.; 5 Division of Infectious Diseases, Department of Medicine, Massachusetts General Hospital, Boston, Massachusetts, USA.; 6 Department of Medicine, University of Pittsburgh School of Medicine, Pittsburgh, Pennsylvania, USA.; 7 Center for Biostatistics in AIDS Research, Department of Biostatistics, Harvard T.H. Chan School of Public Health, Boston, Massachusetts, USA.; 8 Department of Medicine, University of North Carolina at Chapel Hill School of Medicine, Chapel Hill, North Carolina, USA.; 9 The AIDS Clinical Trials Group A5321 Team is detailed in Supplemental Acknowledgments.

**Keywords:** AIDS/HIV, Immunology, T cells

## Abstract

Antiretroviral therapies (ARTs) abrogate HIV replication; however, infection persists as long-lived reservoirs of infected cells with integrated proviruses, which reseed replication if ART is interrupted. A central tenet of our current understanding of this persistence is that infected cells are shielded from immune recognition and elimination through a lack of antigen expression from proviruses. Efforts to cure HIV infection have therefore focused on reactivating latent proviruses to enable immune-mediated clearance, but these have yet to succeed in reducing viral reservoirs. Here, we revisited the question of whether HIV reservoirs are predominately immunologically silent from a new angle: by querying the dynamics of HIV-specific T cell responses over long-term ART for evidence of ongoing recognition of HIV-infected cells. In longitudinal assessments, we show that the rates of change in persisting HIV Nef-specific responses, but not responses to other HIV gene products, were associated with residual frequencies of infected cells. These Nef-specific responses were highly stable over time and disproportionately exhibited a cytotoxic, effector functional profile, indicative of recent in vivo recognition of HIV antigens. These results indicate substantial visibility of the HIV-infected cells to T cells on stable ART, presenting both opportunities and challenges for the development of therapeutic approaches to curing infection.

## Introduction

The needs for both a vaccine and a cure for HIV are underscored by the ongoing impact of this pandemic, which continues to cause close to 800,000 deaths annually (1). Antiretroviral therapy (ART) is capable of durably suppressing HIV replication and halting disease progression — for those able to access and adhere to these regimens. Infection persists, however, in reservoirs of CD4^+^ T cells, and potentially other cell types (2–4), with integrated proviruses that reseed systemic replication if ART is interrupted (2, 5–10). These proviruses often exist in a latent state, characterized by limited transcription and — presumably — a lack of antigen production. This gives rise to one of the central tenets in the study of HIV persistence, which postulates that the persistent reservoir (often called the “latent reservoir”) is not detected by the immune system in individuals on long-term ART. It follows that engaging the immune system to reduce HIV reservoirs depends upon latency reversal to reexpose the immune system to HIV antigen — the “kick and kill” (or “shock and kill”) strategy (11).

Although latency undoubtedly diminishes immune recognition of viral reservoirs, several lines of evidence cast doubt on whether this is absolute in vivo, which would implicate additional contributors to viral persistence (12). Most notably, unspliced, and sometimes multiply spliced, HIV transcripts are readily detectable in PBMCs of individuals on durable ART (13, 14). These observations have recently led some to propose amendments to the “latent reservoir” model, by introducing the idea of a continuum ranging from “deep latency” (no RNA produced) through to an “active reservoir” (15, 16). A key unresolved question, however, is whether these transcripts result in HIV protein production, thus enabling immune recognition. Multiple factors limit the degree to which this can be inferred from direct measures of in vivo viral expression, including sampling difficulties — given that expression may be anatomically or temporally restricted — and the lack of equivalence between readily measurable features (e.g., viral RNA) and bona fide antigen presentation (17, 18). We therefore hypothesized that some level of antigen recognition by HIV-specific T cells may occur in vivo in ART-suppressed individuals with undetectable viremia. We predicted that this would be reflected in relationships between the long-term dynamics of HIV-specific T cell responses and measures of virologic persistence, including frequencies of infected cells as measured by HIV DNA.

Although the T cell response to HIV infection has been generally well characterized, and is known to decay rapidly in the months after ART initiation (19–21), there is a lack of well-powered studies that have addressed the long-term dynamics of these responses in association with virologic parameters while on durable ART. In a previous cross-sectional study, we observed a modest correlation between the magnitudes of T cell responses to the HIV Nef protein with residual frequencies of infected cells (22), providing some initial suggestion that these responses may be maintained by antigen recognition. However, a recent longitudinal study reported that although HIV-specific T cell responses are highly stable on durable ART, no correlations were observed between response magnitudes and intact inducible reservoir size as measured by quantitative viral outgrowth assays across 18 individuals (23). The current study builds upon these earlier reports by uniquely assessing T cell response dynamics over almost 3 years in association with multiple measures of viral persistence, in a cohort of 49 individuals on well-documented sustained ART. Using the scalable IFN-γ enzyme-linked immune absorbent spot (ELISPOT) assay, we confirm that HIV-specific T cell responses persisted over years of well-documented suppressive ART. Strikingly, the persistent T cell responses to the HIV Nef protein (as measured by slopes of change) over 144 weeks were strongly and uniquely associated with the frequencies of infected cells that persisted on ART (as measured by HIV DNA), and these responses disproportionately exhibited a cytotoxic effector functional profile, indicative of recent in vivo antigen recognition. These results provide strong support for ongoing interactions between the immune system and the HIV reservoir over years of ART, with implications for both understanding HIV persistence and designing interventions aimed at curing infection.

## Results

### HIV-specific T cell responses were readily detectable despite long-term ART.

**Figure 1:**
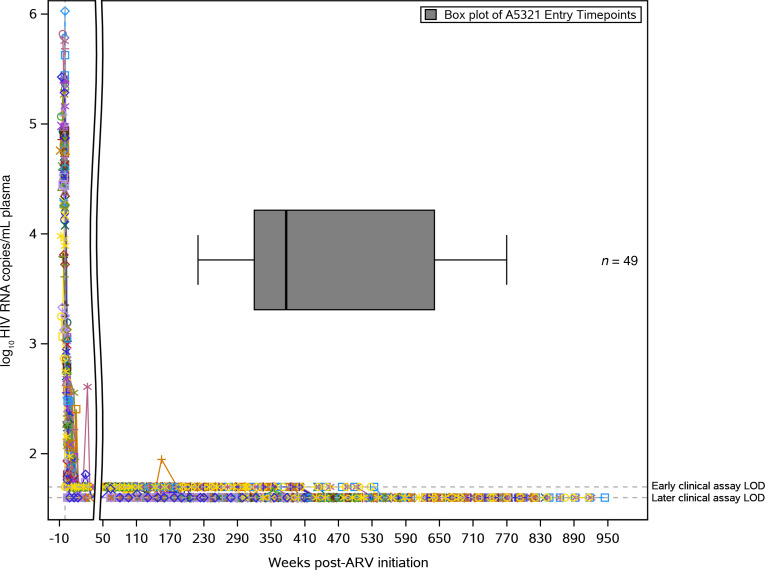
ACTG A5321 cohort participants achieved viral suppression prior to study entry and maintained viral suppression throughout the study period. Log_10_ plasma HIV RNA (copies/mL) by clinical commercial assays for ACTG A5321 cohort study participants included in this longitudinal substudy (*n* = 49), followed from pre-ART initiation (ART initiated in other ACTG trials) through to the A5321 study 168-week time point. Limit of detection for early clinical assays was 50 copies/mL, and for later clinical assays 40 copies/mL. Colored lines represent individual participants, with symbols indicating each clinical viral load measurement. The *x* axis break shows time after ART initiation when all participants achieved initial viral suppression. Box plot shows the distribution of participants’ A5321 study entry time points relative to weeks after ART initiation (minimum, Q1, median, Q3, maximum). ACTG, AIDS Clinical Trials Group; ARV, antiretroviral therapy.

**Figure 2:**
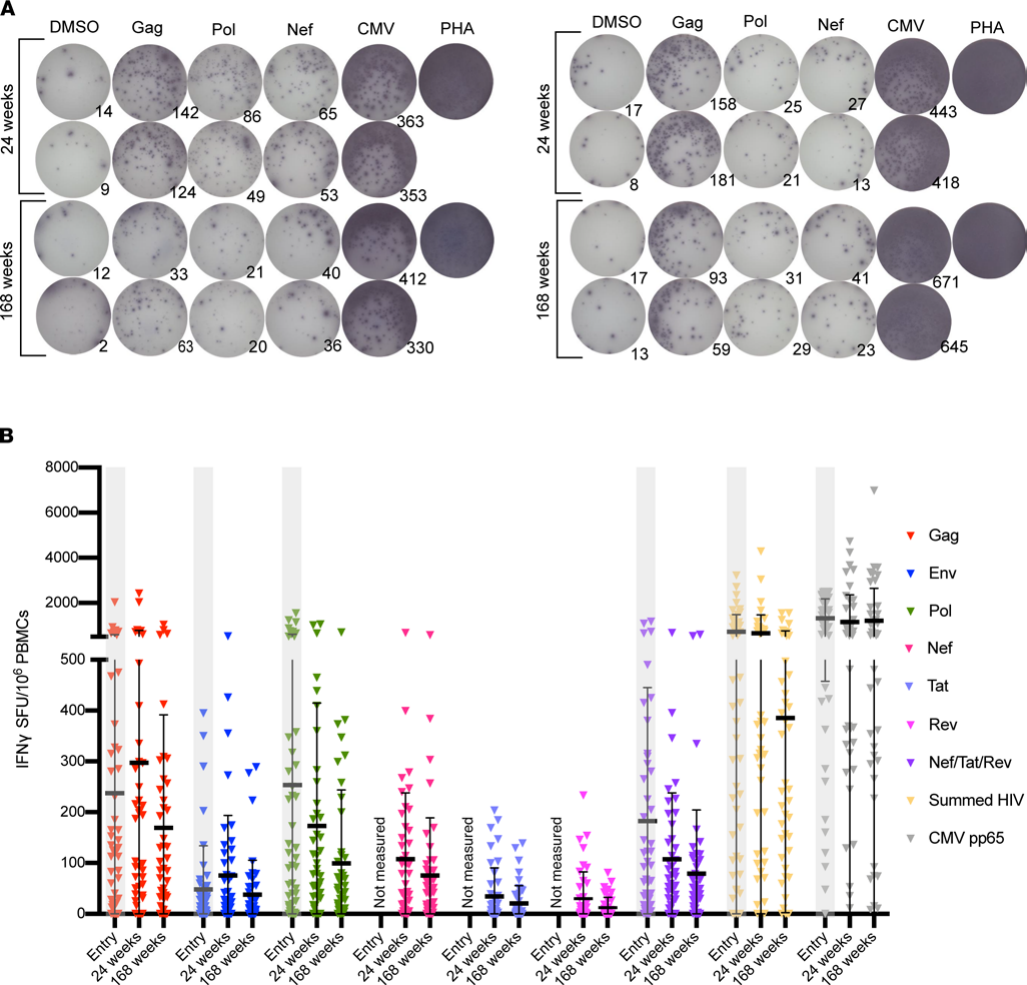
HIV-specific T cell responses are readily detectable ex vivo and persist on long-term ART, primarily directed against HIV Gag, HIV Pol, and HIV Nef. (**A**) Representative IFN-γ ELISPOT results for 2 participants for both time points, with 2 × 10^5^ PBMCs/well. (**B**) Magnitudes of IFN-γ responses are shown for 3 on-ART time points for *n* = 49 participants. Study entry time point data is shaded in gray because it was not performed in batch with 24-week and 168-week time points. Each data point represents the mean SFU/10^6^ PBMCs following background subtraction of negative control wells (duplicates). Vertical lines and error bars represent the mean and standard deviation for each gene product peptide pool. ELISPOT, enzyme-linked immune absorbent spot.

We previously assessed HIV-specific T cell responses in participants from the AIDS Clinical Trials Group (ACTG) A5321 HIV Reservoirs Cohort Study (22), which consists of participants who initiated ART during chronic HIV infection and had subsequent well-documented, sustained virologic suppression (undetectable by clinical assay prior to and throughout the study period) (24). In our prior publication, PBMC samples from A5321 participants were assayed by IFN-γ ELISPOT at study entry, a median of 7 (range 4–15) years after ART initiation (22); here, we extend these results with batched analysis of samples from 24 weeks and 168 weeks after study entry in a subset of 49 participants (Figure 1, Table 1, and Table 2). These ELISPOT assays were performed using whole gene product pools composed of overlapping 15-mer peptides spanning (a) Gag, (b) Env, (c) Pol, (d) Nef, (e) Tat, (f) Rev, (g) Nef/Tat/Rev (combined pool), and (h) cytomegalovirus (CMV) pp65 (control). In our prior study, as well as in the current study, responses against whole gene product peptide pools were background subtracted (thus, nonzero responses were more than 1 time background), but no other ad hoc empirical cutoff was applied — consistent with other studies examining correlations with objectively reported T cell responses as assessed by ELISPOT assay (25). In the current study, IFN-γ–producing HIV-specific T cell responses were readily detected against Gag, Pol, and Nef, with mean, background-subtracted magnitudes at 24 weeks: 297.4, 173.1, and 107.7 spot forming units (SFU) per 10^6^ PBMCs, respectively; and at 168 weeks: 169.2, 99.5, and 75.4 SFU/10^6^ PBMCs, respectively (Figure 2, A and B, and Supplemental Table 1; supplemental material available online with this article; https://doi.org/10.1172/jci.insight.142640DS1).

Between this 24-week and 168-week period, time-averaged responses against Gag were the highest and significantly greater than responses to Env, Nef, Tat, and Rev (all *P* < 0.05) (Supplemental Table 2). Notably, T cell responses directed against Tat and Rev were the lowest in magnitude and negligible at both time points (Figure 2B, Supplemental Table 1, and Supplemental Table 2). The long-term persistence of HIV-specific T cell responses — primarily directed against Gag, Pol, and Nef — over years of ART thus provided initial support for these HIV-specific T cells continuing to interact with antigen.

### Dynamics of Nef-specific T cells correlated with virologic parameters on long-term ART.

**Figure 3:**
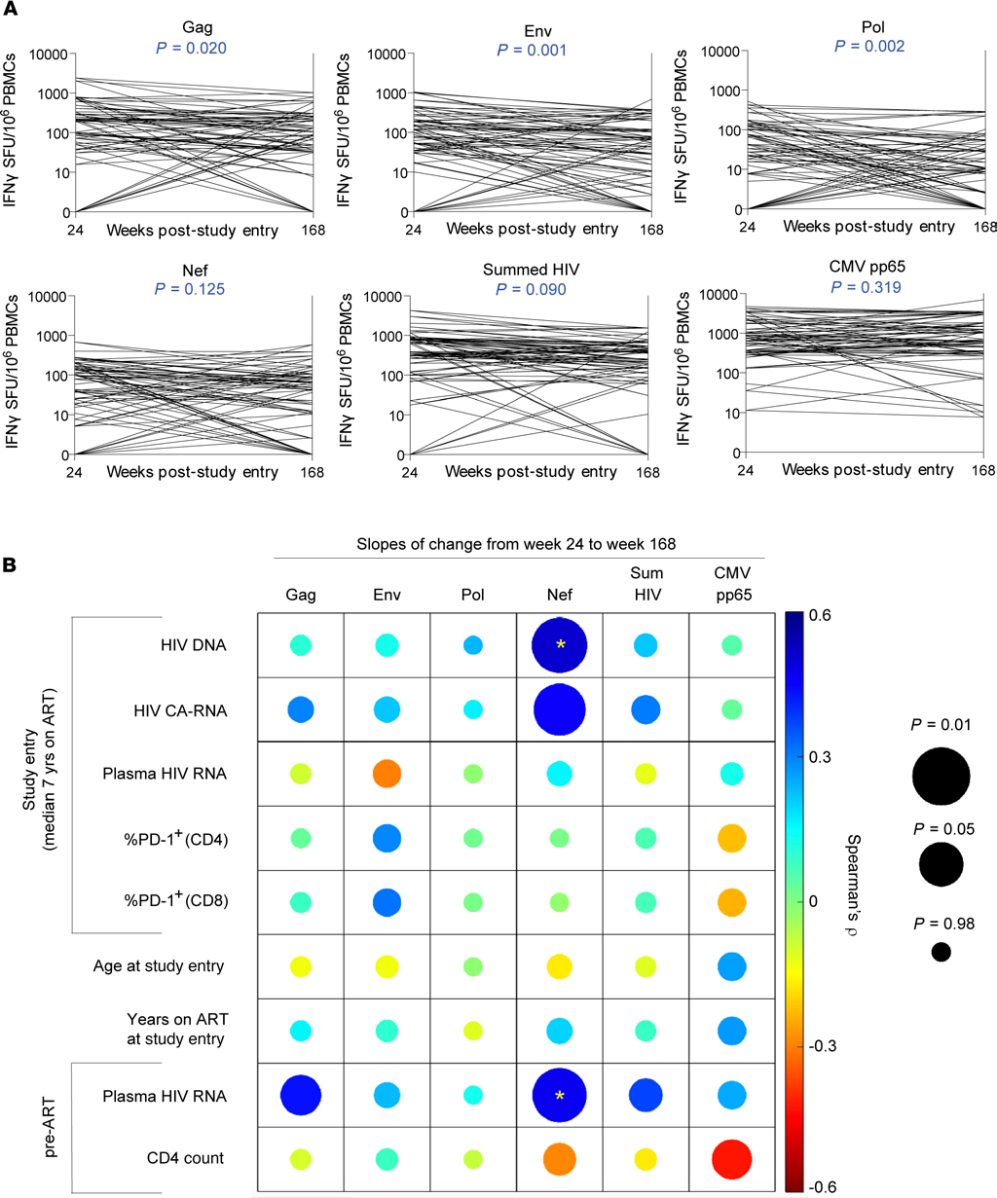
HIV-specific T cell responses are highly stable on long-term ART, with HIV Nef-specific response dynamics uniquely associated with reservoir measures. (**A**) Participant-specific slopes of change in T cell responses from week 24 to week 168 after study entry. *P* values represent the significance level for the covariate time (in weeks) in linear mixed-effects models from Supplemental Table 3. (**B**) Correlogram depicting Spearman’s correlations between slopes of change in raw magnitudes of T cell responses (from panel **A**) with virologic and immunologic parameters. Color scale bar represents magnitude of correlation coefficient. Circle size represents unadjusted *P* values, corrected for the false discovery rate. Asterisks represent adjusted *P* values: for HIV DNA controlling for pre-ART plasma HIV RNA, pre-ART CD4^+^ T cell count, and years on ART at study entry, and pre-ART plasma HIV RNA controlling for HIV DNA at study entry; all adjusted *P* values are corrected for the false discovery rate (*P < 0.05).

To further characterize the long-term dynamics of HIV-specific T cell responses in A5321 cohort participants on durable ART, we categorized participants’ IFN-γ ELISPOT responses with data from batched analysis of samples from 24 weeks and 168 weeks after study entry as increasing, decreasing, or not changing (predefined as ≤15% change) and observed considerable heterogeneity (Supplemental Figure 1). Notably, population-average responses to Nef, summed HIV, and CMV pp65 did not decline significantly over this 144-week time period, whereas responses to Gag, Env, and Pol all showed significant declines over time (Figure 3A and Supplemental Table 3). However, all HIV-specific T cell responses demonstrated remarkable persistence, with the responses that showed a significant decline averaging only between 0.35% to 0.62% loss per week in IFN-γ ELISPOT assays (Supplemental Table 3).

To determine whether ongoing antigen recognition by HIV-specific T cells could be maintaining IFN-γ–producing HIV-specific T cell responses, we next examined associations between the slopes of change of T cell response magnitudes between 24 weeks and 168 weeks after study entry (based on absolute changes on a linear scale) with on-ART virologic parameters, including total cell-associated HIV DNA (CA-DNA), cell-associated HIV RNA (CA-RNA), and plasma HIV RNA by integrase single-copy assay (iSCA). Due to a large number of statistical tests, we corrected for the false discovery rate at a stringent level of 0.05; additionally, we controlled for potential confounders in associations. The dynamics of responses to Nef were uniquely significantly associated with pre-ART viral loads before and after adjusting for CA-DNA at study entry (adjusted *r* = 0.48, *P* = 0.012; Figure 3B and Supplemental Table 4), despite participants having been on ART for over a median of 7 years when responses were first measured. Strikingly, the slopes of change in Nef-specific responses were also unique in exhibiting highly significant direct associations with any on-ART virologic parameter after adjusting for potential confounding by pre-ART plasma viral load, pre-ART CD4^+^ T cell count, and years on ART at study entry — specifically, on-ART CA-DNA at study entry (adjusted *r* = 0.50, *P* = 0.012). Slopes of change in Nef-specific responses were also uniquely associated with CA-RNA at study entry (*r* = 0.46, *P* = 0.018), though significance did not survive adjustment for confounders (adjusted *r* = 0.40, *P* = 0.063) (Figure 3B and Supplemental Table 4). These results indicate that higher frequencies of persistent infected cells (CA-DNA), and, potentially, higher levels of viral transcription (CA-RNA), were associated with greater maintenance of Nef-specific responses, consistent with some ongoing stimulation by infected cells. Slopes of change in HIV-specific T cell responses were not associated with PD-1 levels on total CD4^+^ or CD8^+^ T cells, nor were they associated with either age at study entry or years on ART at study entry (Figure 3B and Supplemental Table 4), though they generally correlated with each other (Supplemental Table 5). Analyzing slopes of change in log_10_-transformed T cell response magnitudes, reflecting proportional changes in responses rather than absolute changes, revealed a significant association only between the dynamics of Nef-specific T cell responses with on-ART CA-DNA at study entry (adjusted *r* = 0.49, *P* = 0.029; Supplemental Table 6). Proportional changes in HIV-specific responses generally correlated with each other (Supplemental Table 7). Thus, whether dynamics were measured on an absolute or proportional change scale, Nef-specific response persistence was uniquely associated with HIV-infected cell frequencies.

To rule out the potential for IFN-γ–producing Nef-specific ELISPOT responses being enriched in low-magnitude false-positive responses, thereby influencing results, we performed a sensitivity analysis applying a rigorous positivity cutoff of more than 3 times background. Data from a given participant were excluded if the results from both of the time points fell below this threshold (since going from detectable to below the threshold or vice versa are still biologically meaningful). Applying this cutoff, Nef-specific responses still exhibited remarkable stability, with no significant decline over time (Supplemental Figure 2 and Supplemental Table 8). In correlation analyses, the magnitudes of correlations between the slopes of change in Nef-specific T cell responses with on-ART virologic parameters (CA-DNA and CA-RNA) were strengthened, relative to those observed without applying this cutoff (Supplemental Table 9). Altogether, these results suggest that Nef-specific T cell responses were preferentially maintained by ongoing interactions with HIV-infected cells, though all responses were likely maintained to some extent by ongoing HIV antigen recognition given their exceptional persistence.

### Nef-specific T cells exhibited functional profiles consistent with recent antigen recognition.

**Figure 4:**
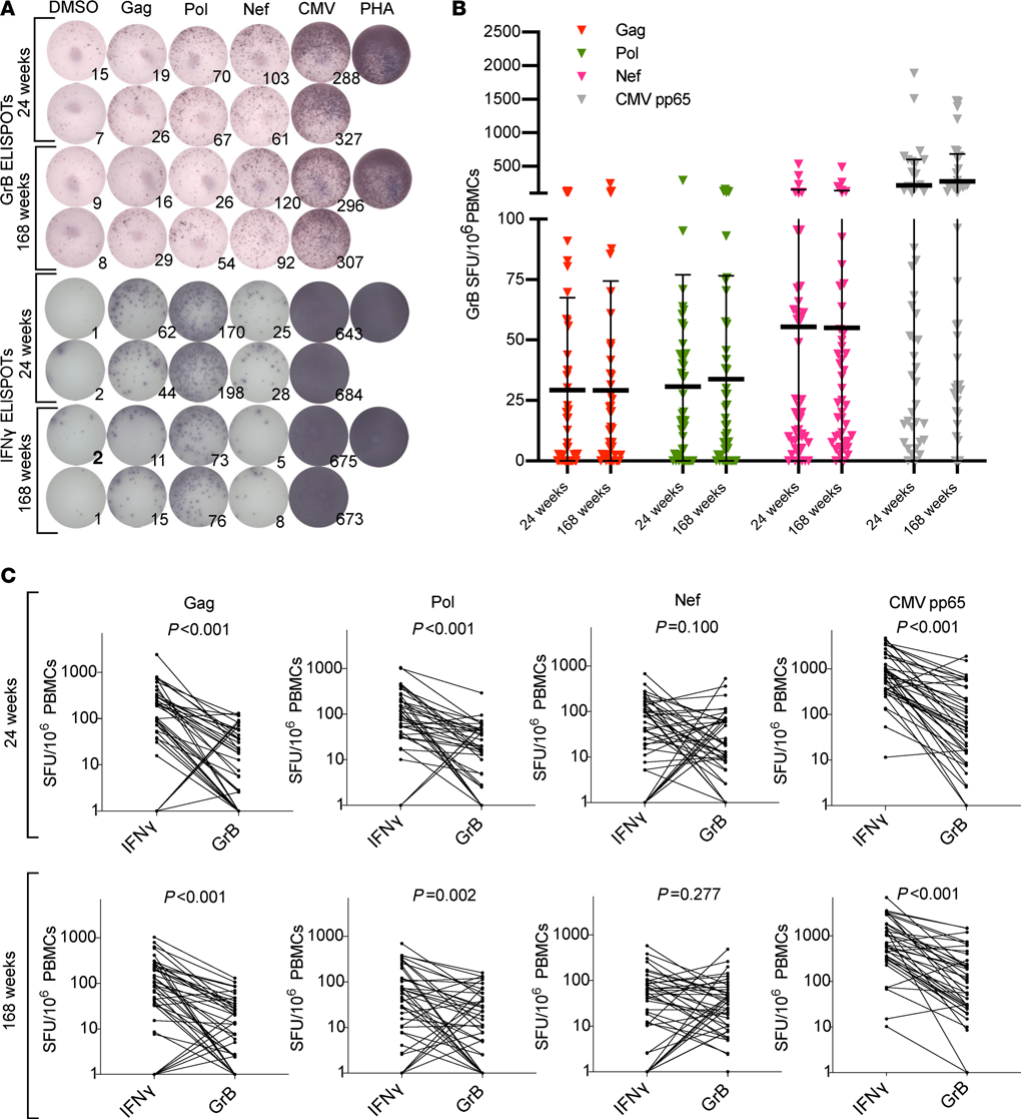
HIV-specific T cells demonstrate cytotoxic ability, preferentially directed toward HIV Nef, evidencing recent in vivo antigen exposure. (**A**) Corresponding granzyme B (GrB) and IFN-γ ELISPOT results for 1 participant at both time points, with 2 × 10^5^ PBMCs/well. (**B**) Magnitudes of granzyme B responses are shown for 2 batched on-ART time points for *n* = 49 participants. Each data point represents the mean number of SFU/10^6^ PBMCs following background subtraction of mean of negative control wells. Vertical lines and error bars represent the mean and standard deviation for each gene product peptide pool. (**C**) Pairwise comparisons of granzyme B responses versus IFN-γ responses for Gag, Pol, Nef, and CMV pp65 at both time points. *P* values calculated by Wilcoxon matched pairs signed-rank test.

We next investigated whether the functional properties of HIV-specific CD8^+^ T cells would yield insights into their recent histories of in vivo antigen encounter. Data from human studies and animal models have highlighted ex vivo granzyme B production as a distinguishing feature of virus-specific effector CD8^+^ T cells that have recently encountered antigen in vivo, through either infection or vaccination (26–30). Although granzyme B production can be induced in memory CD8^+^ T cells, this requires more than 24 hours of in vitro stimulation, whereas IFN-γ is produced rapidly from both memory and effector CD8^+^ T cells (26, 31, 32). Thus, ex vivo ELISPOT measurements of granzyme B have been established as an “immune diagnostic” means of identifying effector responses to active infections (31, 33). To quantify the effector functionalities of HIV-specific T cells on long-term ART, we performed batched granzyme B ELISPOT assays on week 24 and week 168 samples (Figure 4A). We focused on the Gag, Pol, and Nef peptide pools, having observed these to be the most immunogenic by IFN-γ ELISPOT. Overall, granzyme B–producing HIV-specific responses were substantially lower in magnitude than IFN-γ responses (Figure 4, B and C, and Supplemental Table 1). The mean, background-subtracted magnitudes of granzyme B responses relative to each other were Nef>Pol>Gag (at both time points; Figure 4B and Supplemental Table 1), contrasting with IFN-γ: Gag>Pol>Nef (Figure 2B and Supplemental Table 1). As with IFN-γ, categorizing participants’ granzyme B responses as increasing, decreasing, or not changing revealed heterogeneity (Supplemental Figure 3), though proportionally there were fewer decreasing responses, and the population-average levels of granzyme B responses were highly stable over time to all HIV gene products (Figure 4B and Supplemental Table 3). In contrast to IFN-γ, we did not observe any significant correlations between the slopes of change of granzyme B responses with virologic measures of HIV persistence (Supplemental Table 10 and Supplemental Table 12). These results may reflect the additional complexity that whereas both IFN-γ–producing and granzyme B–producing cells can be maintained by infected cells producing antigen, the latter are more likely to also perturb the virologic measures by eliminating infected cells (34).

To further assess the functional profiles of HIV-specific T cell responses, we performed pairwise comparisons of granzyme B responses versus IFN-γ responses for each of the gene products tested (Figure 4C). At both time points, granzyme B response magnitudes to Gag, Pol, and CMV pp65 were substantially lower than IFN-γ responses (all *P* < 0.05) (Figure 4C). Contrasting this, the magnitudes of granzyme B responses versus IFN-γ responses to Nef were not significantly different from each other at either time point (*P* = 0.100 on week 24, *P* = 0.277 on week 168). These data indicate that in addition to being preferentially maintained over time, T cell responses directed against the early HIV gene product Nef disproportionately exhibit effector functional profiles compared with the late gene products Gag and Pol (though granzyme B responses to these late gene products were still detected). Persistent HIV-specific granzyme B responses are indicative of recent antigen encounter, supporting the hypothesis that there is in vivo stimulation by HIV-infected cells despite suppressive ART.

The results showing that CMV pp65–specific T cells exhibited lower IFN-γ/granzyme B ratios compared with Nef-specific responses were somewhat unexpected, given that CMV infection is characterized by episodic low-level antigen exposure due to stochastic reactivation (35). Although our study hypothesized a similar scenario for HIV-infected cells, we had assumed that this would occur to a greater degree for CMV, driving more of an effector phenotype. Given that CMV-specific responses in HIV-coinfected individuals can have a robust CD4^+^ component (36), one explanation could be that this lesser ratio is the result of greater representation of CD4^+^ versus CD8^+^ T cells in the CMV versus Nef responses (with less granzyme B from the CD4^+^ T cells) (37). To test this, we performed paired IFN-γ and granzyme B ELISPOTs on PBMCs, CD8-depleted samples, and CD4-depleted samples in a subset of 6 participants, with depletions achieving substantial reductions in respective cell amounts (Supplemental Figure 4A). The results indicate that both CMV pp65 and HIV Nef responses were predominately CD8^+^ T cell driven (Supplemental Figure 4B). Having ruled out a skewing toward a CD4^+^ T cell response as a major driver in the higher IFN-γ/granzyme B ratio for CMV pp65–specific versus HIV Nef-specific responses, we suggest that this observation may reflect greater or more frequent antigen exposure of HIV Nef versus CMV pp65.

## Discussion

An important aspect of how HIV persists in individuals on long-term ART is through the evasion of immune recognition, predominately thought to be achieved through the maintenance of strict viral latency, with an additional aspect of anatomical sequestration. This perception that the reservoir is entirely latent has begun to shift lately, in response to both a new understanding of the dynamic nature of the HIV reservoir (driven by the clonal expansion of infected cells) and new insights into ongoing viral transcriptional activity on ART (16, 38). To date, however, this has yet to prompt widespread reconsideration of the relationship between the HIV-specific T cell response and the HIV reservoir. The current study provides evidence that challenges the prevailing model of a lack of reservoir immune surveillance, by indicating a level of ongoing antigenic stimulation of HIV-specific T cells in ART-suppressed individuals. Nef-specific T cells stood apart from those of other HIV gene products in this regard, supporting that early gene products (Nef, Tat, and Rev, of which only Nef was appreciably immunogenic, as was also seen in other studies; refs. 23, 39) have lower thresholds to expression in a reactivation setting compared with late gene products (Gag, Pol, and Env), which are expressed only after a cell has built up sufficient levels of Rev to drive nuclear export of unspliced and singly spliced viral transcripts (40, 41). The preferential maintenance of Nef-specific T cells was presented as a hypothesis of the current study based on both this conceptual model and our previous observation that Nef-specific T cells recognized cells reactivated from an in vitro latency model prior to recognition by Gag-specific T cells, or detectable Gag expression (22). However, we also note the potential role of defective HIV proviruses in our observations, both in general, as a source of antigenic stimulation on ART, and through their potential to contribute to the unique relationship between total HIV DNA and Nef-specific T cell responses. We have previously demonstrated that a subset of defective proviruses can give rise to antigen expression that can be recognized by HIV-specific T cells (42), in line with other studies that have reported protein expression (43, 44). Of particular interest was a study that demonstrated that a defective provirus with a 2.4 kb internal deletion was nonetheless capable of producing Gag and Nef proteins (44). An alternative plausible explanation for our observations is that some antigens (e.g., Nef) may be more likely to be expressed from defective proviruses than others, driving the relationship with total HIV DNA. Our data do not provide direct insights into the relative roles of intact versus defective proviruses in maintaining HIV-specific T cell responses on ART, but we suggest that this will be an interesting area for future investigation.

The dynamics of T cell responses to other viruses elicited through vaccination or infection may be instructive toward understanding the persistence of HIV-specific T cell responses. Exposure to transient antigen through vaccination with live yellow fever virus (YFV) induces an initial, robust, virus-specific effector T cell response, characterized by the expression of effector molecules such as granzyme B (45, 46). YFV-specific T cells persist long after viral RNA is undetectable, capable of expressing IFN-γ years after vaccination despite the lack of ongoing YFV antigen expression (45, 46). Unlike short-lived YFV-specific effector T cells, however, this separate population of long-lived YFV-specific T cells are incapable of expressing granzyme B without ongoing antigen stimulation (46). In contrast, CMV, or the murine equivalent, MCMV, is considered a “smoldering” infection characterized by persistent, low-level viral antigen expression interrupted by intermittent productive viral reactivation events (35, 47). CMV infection induces a robust, long-lived, CMV-specific T cell response, capable of expressing both IFN-γ and granzyme B, reflecting ongoing exposure to CMV-infected cells expressing antigen (39). Our data demonstrating that HIV-specific T cells persist with an effector functional profile (capable of producing granzyme B) suggest that, like CMV-specific T cells and unlike YFV-specific T cells, HIV-specific T cells periodically encountered their cognate antigens. Though additional studies are warranted, our data showing that the Nef-specific T cell response exhibited a proportionally greater effector functional profile than the CMV-specific T cell response additionally raise the possibility that Nef-specific T cells encounter Nef antigen either to a greater extent or more frequently than CMV pp65–specific T cells encounter their antigen.

Do our results allow for any inferences into how frequently infected cells are recognized by HIV-specific T cells in vivo? Although numerous aspects of complexity introduce caveats to such an analysis (e.g., tissue distributions), our data do allow for side-by-side comparisons between the peripheral blood frequencies of infected cells with antigen expression potential and those of HIV-specific T cells, which may be informative. The mean frequency of Nef-specific T cells on week 24 of our study was 107.7/10^6^ PBMCs, whereas the median total frequency of HIV-infected cells (CA-DNA) was 515.7/10^6^ CD4^+^ T cells (on week 0 — this was not measured on week 24, but CA-DNA is highly stable on long-term ART; ref. 48) or roughly 103/10^6^ PBMCs. These infected cells, however, predominately contain defective proviruses (49), many of which are likely incapable of expressing antigens (42). It can therefore be reasonably estimated that, in most individuals, Nef-specific T cells are at least as frequent as infected cells with the potential to express antigen. Our data indicating that the former are influenced by the latter therefore suggest that antigen expression was more likely to be a common versus a rare event in vivo among infected cells with this potential. However, further study is needed, and characterizing the clonal dynamics of HIV-specific T cells on ART may yield additional insights.

Although latency almost certainly contributes to viral persistence, our findings indicating that HIV reservoirs were not fully hidden from circulating cytotoxic T cells raise the question of what additional mechanisms may be at play. We first consider the role of immune escape, the process by which HIV evades recognition by acquiring mutations in T cell epitopes. Immune escape plays a critical role in limiting the overall efficacy of the HIV-specific T cell response in untreated infection, and HIV reservoirs show clear evidence of past selection, in the form of extensive sequence variation in known T cell epitopes (50). However, the question at hand pertains to HIV-specific T cell responses that show evidence of being maintained by recent antigen recognition, indicating that they target epitopes that are intact in at least a portion of the reservoir. Further supporting this idea are the previous observations that (a) the fixation of escape mutations leads to the contraction of corresponding T cell responses (51) and (b) the substantial majority of HIV-specific T cells that remain detectable after years of ART target epitopes for which escape is not fixed in corresponding reservoir viruses (52, 53). As with latency, our data do not lead us to contest the idea that the fixation of escape mutations in the reservoir diminishes the overall potential for immune recognition or the value of therapeutic strategies to address either of these limitations. However, we are still left with the question of how to reconcile our findings indicating an appreciable level of ongoing in vivo recognition of infected cells by cytotoxic (granzyme B–producing) T cells, with the overall stability of HIV reservoir sizes.

We therefore draw from 2 recent findings in the field to propose how an HIV reservoir may persist without being fully hidden from circulating cytotoxic T cells. The first derives from the recent demonstrations that the HIV reservoir is predominately composed of infected T cells that have undergone clonal expansion (54–58), with different clones dynamically “waxing and waning” over time (56). Thus, HIV-specific T cells may frequently eliminate infected cells, only to have these replaced by clonal expansion of other reservoir-harboring cells. There have been somewhat conflicting recent reports regarding this possibility — from groups that approached the question from different angles — highlighting the need for further study (42, 59, 60).

Second, we have recently reported that reservoir-harboring cells exhibit intrinsic resistance to T cell–mediated elimination (61), mediated in part by Bcl-2 overexpression, which antagonizes perforin/granzyme killing (62). In fact, although it has been generally assumed in our field that the encounter between an antigen-expressing HIV-infected cell and a functional (e.g., perforin/granzyme releasing) CD8^+^ T cell will result in elimination, this overlooks the role of the target cell as an active partner in the killing process. Multiple regulatory mechanisms exist, in both physiological and pathological states, by which target cells determine whether to undergo apoptosis, despite receiving a perforin/granzyme hit (63–65). Thus, one way to resolve our findings with others in the field is to propose that the recognition of HIV-infected cells by HIV-specific cytotoxic T cells may occur with some frequency in vivo but that this often does not result in target cell elimination. An intriguing possibility is that the combined effects of selection, based on intrinsic susceptibility to CD8^+^ T cells, and clonal expansion of surviving cells may enable the evolution of a resistant reservoir, paralleling the phenomenon of “immunoediting” in cancer (12). Although latency reversal will likely be a critical component of curing HIV infection, our findings raise the hypothesis that — in lieu of an ideal latency-reversing agent — reductions in HIV reservoirs may be achievable by boosting immune targeting of existing expression of early gene products (such as Nef, and in a manner that targets nonescaped epitopes) while enhancing cytotoxic function, limiting clonal expansion, and addressing resistance to cytotoxic T cells in reservoir-harboring cells.

## Methods

### Study design.

Data for this manuscript were collected on a longitudinal cohort of participants who initiated ART during chronic HIV infection in ACTG trials for treatment-naive individuals and enrolled in the ACTG HIV Reservoirs Cohort Study (A5321) (24). A5321 cohort participants were recruited from 17 clinical research sites in the United States through the ACTG network. IFN-γ ELISPOTs were previously performed using samples from 99 participants at A5321 study entry (22), and a subset of 49 participants were selected from the original 99 for this longitudinal substudy based on sample availability. All gene products and negative controls were tested in duplicate, with 1 replicate of phytohemagglutinin-positive (PHA-positive) control. Assays performed under these same conditions have been previously validated in other participant cohorts. Outliers were not defined or excluded. Participants in the current substudy were followed up at least every 6 months after study entry, with documented sustained viral suppression (plasma HIV RNA levels < 50 copies/mL by commercial assays starting on week 48 on ART and at all subsequent time points; Figure 1). One participant had a large viral blip (>1000 copies/mL) 43 weeks prior to their 168-week A5321 study time point, and data were right-censored for this participant after the 24-week A5321 study time point. Clinical data and paired plasma and PBMC samples were available from pre-ART and on-ART study visits. We measured HIV levels (CA-DNA, CA-RNA, and plasma iSCA) and PD-1 levels (on CD4^+^ and CD8^+^ cells) on samples obtained at A5321 study entry (median 7 years on ART), and plasma HIV RNA levels and CD4^+^ T cell counts were obtained from pre-ART clinical data. One participant later revoked consent for further testing and was excluded from analysis. We hypothesized a priori that the long-term dynamics of T cell responses to the early HIV gene product Nef (measured by IFN-γ ELISPOT) would be associated with infected cell frequencies as measured by HIV DNA.

### Virologic assays.

HIV CA-DNA and CA-RNA were measured by quantitative PCR (qPCR) assays in PBMCs using previously described methods (66). CA-DNA and CA-RNA values per million CD4^+^ T cells were calculated by dividing the total CA-DNA or CA-RNA copies/million PBMCs (normalized for CCR5 copies measured by qPCR as published in ref. 66) by the CD4^+^ T cell percentage (× 0.01) reported from the same specimen date or from a CD4^+^ T cell percentage imputed using linear interpolation from specimen dates before and after the CA-DNA or CA-RNA results. Cell-free HIV RNA was quantified by iSCA in blood plasma (5 mL) (67).

### Immunologic assays.

PBMCs obtained at A5321 study entry were stained with the following monoclonal antibodies to evaluate surface PD-1 expression: CD3 APC-H7 (catalog 560176), CD4 PC5 (catalog 555348), CD8 V450 (catalog 560347), PD-1 (clone M1H4), A488 (catalog 557860) (all from BD Biosciences), and LIVE/DEAD Aqua (Invitrogen, Thermo Fisher Scientific). Cells were fixed in 1% paraformaldehyde and analyzed using a BD LSRFortessa (BD FACSDiva software) within 24 hours after staining. Lymphocytes were identified based on size and granularity. The lymphocyte population was filtered through side scatter area versus side scatter height histogram to eliminate doublets from the analysis. Single cells were analyzed using LIVE/DEAD Aqua dye exclusion, and then CD4^+^ and CD8^+^ populations were defined based on dual expression with CD3. These 2 populations were plotted against PD-1. Fluorescence minus one controls were used to define the PD-1^+^ T cell populations.

### Peptide pools.

The following sets of consensus HIV clade B 15 amino acid peptides (overlapping by 11 amino acids) were supplied by the NIH AIDS Research and Reference Reagent Program: Gag (catalog 8117), Env (catalog 9480), Pol (catalog 6208), Tat (catalog 5138), Rev (catalog 6445), and Nef (catalog 5189). All peptides were dissolved at 5 mg/mL in 12.5% DMSO (Corning) and 87.5% PBS (Gibco, Thermo Fisher Scientific). Peptides were pooled into whole gene product peptide pools and adjusted to a final concentration of 20 μg/mL/peptide in PBS. A CMV pp65 PepMix peptide pool (JPT Peptide Technologies) was dissolved separately in DMSO and adjusted to a final concentration of 20 μg/mL/peptide in PBS.

### IFN-γ and granzyme B ELISPOT assays.

Multiscreen IP 96-well PVDF plates (MilliporeSigma) were either directly coated with 100 μL/well of PBS plus 0.5 μg/mL primary anti-human IFN-γ antibody (clone 1-D1K, Mabtech) overnight at 4°C or first primed with 20 μL of 35% ethanol/well, immediately washed 6 times with 200 μL double-distilled H_2_O, and then coated with 100 μL/well of PBS plus 15 μg/mL primary anti-human granzyme B antibody (clone GB10, Mabtech) overnight at 4°C. Granzyme B plates were washed 6 times with 200 μL PBS and blocked with RPMI 10% FBS (Gibco, Thermo Fisher Scientific) (R-10) at 37°C 5% CO_2_. PBMCs were thawed and resuspended in R-10 and added to plates at 100,000–200,000 cells/well. HIV peptide pools (20 μg/mL/peptide) were added at 10 μL/well for a final concentration of 1 μg/mL/peptide in <0.5% DMSO. CMV pp65 peptide pools were added at 10 μL/well for a final concentration of 1 μg/mL/peptide in <0.5% DMSO. PHA was dissolved in DMSO and PBS to 200 μg/mL and then added to a final concentration of 1 μg/mL as a positive control. DMSO (0.5%) in PBS and R-10 media were used as negative controls. Plates were incubated for 18 hours at 37°C with 5% CO_2_. Plates were washed 6 times with 200 μL PBS. Biotinylated secondary IFN-γ antibody (clone 7-B6-1, Mabtech) at 0.5 μg/mL in PBS or biotinylated secondary anti–granzyme B antibody (clone GB11, Mabtech) at 1.0 μg/mL in PBS was added to the plates to a final volume of 100 μL and incubated for 1 hour in the dark. Plates were then washed 6 times with PBS, 0.5 μg/mL of Streptavidin-ALP (Mabtech) was added to IFN-γ plates at 100 μL/well, and 1 μg/mL of Streptavidin-ALP (Mabtech) was added to granzyme B plates at 100 μL/well and incubated for 1 hour. Plates were washed 6 times with PBS, and then color development substrate solution: 10.6 mL of double-distilled H_2_O, 400 μL 25x AP Color Development Buffer (Bio-Rad), 100 μL AP color reagent A (Bio-Rad), and 100 μL AP color reagent B (Bio-Rad) was added to the plate at 100 μL/well for 15 minutes. After removal of the color development substrate solution, 0.5% of Tween-20 in PBS was added at 100 μL/well for 10 minutes. Plates were then washed with water and left overnight to dry. Plates were counted using ImmunoSpot S6 Ultimate Analyzer and ImmunoSpot software (Cellular Technology Limited). As in our prior publication (22), ELISPOT responses against whole gene product peptide pools were background subtracted, but no other cutoff value was applied (the positivity criteria of 1 [>50 SFU/million PBMCs after background subtraction and 2] more than 2 times above background in our prior publication were applied only to the responses for peptide pool matrix mapping).

### CD8-depleted and CD4-depleted PBMC experiments.

Paired granzyme B and IFN-γ ELISPOT assays were performed as described above, with the exception that PBMCs were counted and divided into 3 separate populations after thaw: PBMCs, CD8-depleted PBMCs, and CD4-depleted PBMCs. These separate populations were plated at between 20,000 and 200,000 cells/well. CD8^+^ T cells and CD4^+^ T cells were depleted by positive selection (EasySep, STEMCELL Technologies). Samples were stained with anti-CD8 FITC (RPA-T8), anti-CD4 Pacific Blue (RPA-T4), anti-CD3 BV785 (SK7), anti-CD14 Alexa Fluor 647 (63D3) (all from BioLegend), and LIVE/DEAD Fixable Aqua dye (Thermo Fisher Scientific L34966). Flow cytometry data were collected using an Attune NxT flow cytometer, and data were analyzed using FlowJo.

### Statistics.

Statistical analyses including univariate statistics and Spearman correlations and partial correlations (adjusting for potential confounders) were conducted in SAS University Edition. Correlation results were corrected post hoc for multiple comparisons by the false discovery rate method of Benjamini and Hochberg (68) using the SAS MULTTEST procedure (FDR option). For Supplemental Figure 1 and Supplemental Figure 3, responses were categorized as “not changing” based on a ≤15% change in either direction; this threshold was arbitrarily predefined based on the frequency distribution of the percent change data. Slopes of change in Figure 3B, Supplemental Table 4, Supplemental Table 5, Supplemental Table 10, and Supplemental Table 11 were calculated based on absolute changes on a linear scale between week 24 and week 168 after A5321 study entry, excluding participants who had a change from 0 magnitude to 0 magnitude. Analyses for Supplemental Table 6, Supplemental Table 7, Supplemental Table 12, and Supplemental Table 13 used slopes of change calculated based on proportional changes on a log_10_ scale between week 24 and week 168 after A5321 study entry, excluding participants who had a change from 0 magnitude to 0 magnitude; slopes reflecting a change from 0 magnitude to a nonzero magnitude were analyzed as the highest rank, and slopes reflecting a change from a nonzero magnitude to 0 magnitude were analyzed as the lowest rank. Statistical analyses including 1-way ANOVA, Friedman tests, and Wilcoxon signed-rank tests were conducted in GraphPad Prism v.8.0. Plots for figures were made in GraphPad Prism v.8.0 and SAS University Edition. A custom code was generated in MATLAB v.9.7 to produce the correlogram in Figure 3B. All linear mixed-effects models were conducted using the R “lme4” package (69), with random intercepts only on the participant level modeled for the random effects; multiple comparisons were made where indicated using the R “multcomp” package (70), adjusting for multiple comparisons using Tukey’s all-pair method. Linear mixed-effects models used log_10_-transformed response data to meet normality assumptions, treating 0-valued responses as missing data. Imputation was not used to address missing data, as the degree of missingness was low. All statistical tests were 2-sided, α = 0.05.

### Study approval.

Each ACTG A5321 clinical research site had the A5321 protocol and consent form, and its relevant parental protocols and consent forms, approved by their local IRBs, as well as registered with and approved by the Division of AIDS Regulatory Support Center Protocol Registration Office, prior to any participant recruitment and enrollment. Once a participant for study entry was identified, details were carefully discussed with the prospective participant by clinical staff at the site. The participant (or, when necessary, the parent or legal guardian if the participant was under guardianship) was asked to read and sign the ACTG-approved protocol consent form.

## Author contributions

RBJ designed the study. EMS, ARW, RT, AST, SHH, TRD, ST, JKB, TMM, AD, GQL, AG, PK, WDCA, JCC, and BM performed experiments. EMS, ARW, RT, ST, SHH, JKB, CML, RJB, BM, JCC, and JWM analyzed data. RTG, DKM, JJE, and JWM provided participant data. EMS, ARW, and RBJ wrote the manuscript. All authors contributed to the critical revision of the manuscript. The order of the co–first authors was determined based upon the order in which they began working on the project.

## Supplementary Material

Supplemental data

## Figures and Tables

**Table 1:**
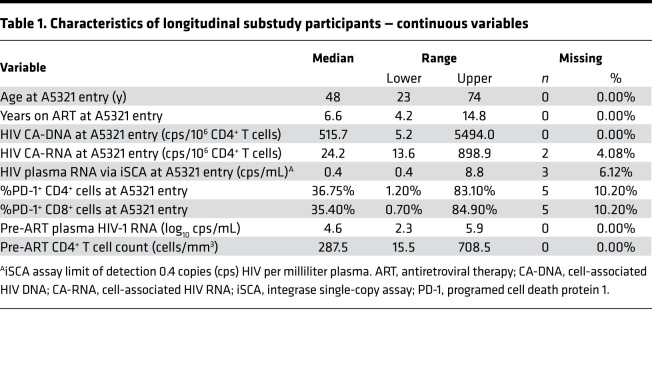
Characteristics of longitudinal substudy participants — continuous variables

**Table 2:**
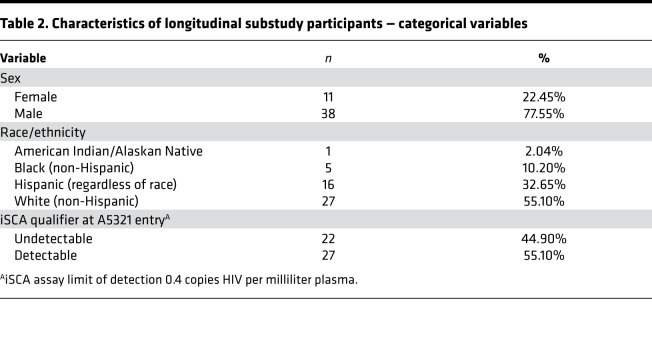
Characteristics of longitudinal substudy participants — categorical variables

## Version 1 01/05/2021

In-Press Preview

## Version 2 02/08/2021

Electronic publication
