# Supplementary material for: HIV-specific T cell responses reflect substantive in vivo interactions with antigen despite long-term therapy
Source: JCI Insight. 2021 Feb 8;6(3):e142640. doi: 10.1172/jci.insight.142640 (PMC7934865; doi:10.1172/jci.insight.142640)
Supplement: Supplemental data [file jciinsight-6-142640-s188.pdf]

Supplementary materials

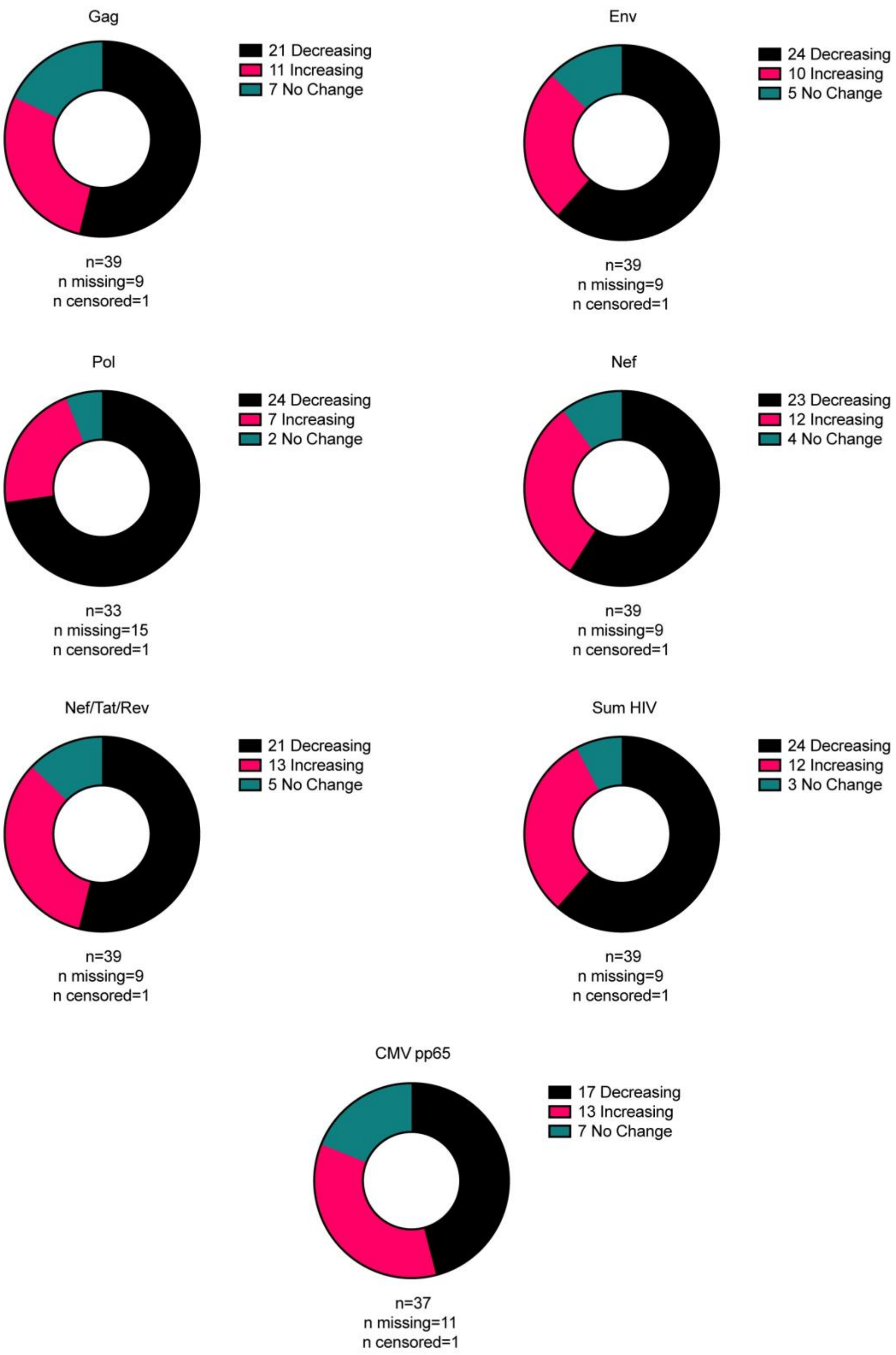

Figure S1. Patterns of longitudinal IFNγ T-cell response changes between 24-168 weeks post-A5321 study entry. Participant T-cell responses were categorized as either increasing, decreasing, or not changing (defined as ≤15% change in either direction) between the two batched on-ART timepoints, and data is shown in parts-of-whole plots. Participants were categorized as missing if they had a missing value for a response at either timepoint. Participants were categorized as censored if they had a censored value for a response at either timepoint.

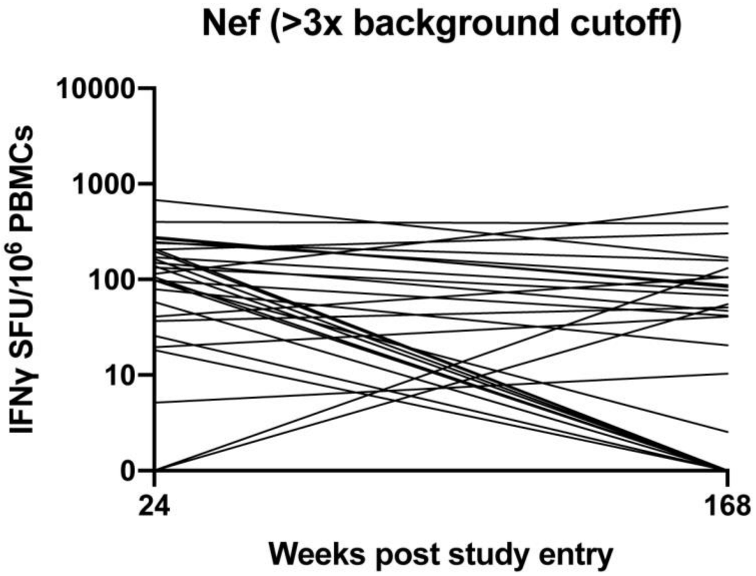

Figure S2. Nef-specific T-cell responses remain highly stable on long-term ART across the cohort when an ELISPOT positivity cutoff of >3x background is applied. Participant Nef-specific T-cell responses were excluded if the results from both the week 24 and week 168 timepoints fell below the >3x background threshold, but were retained if a response at either timepoint was above this threshold.

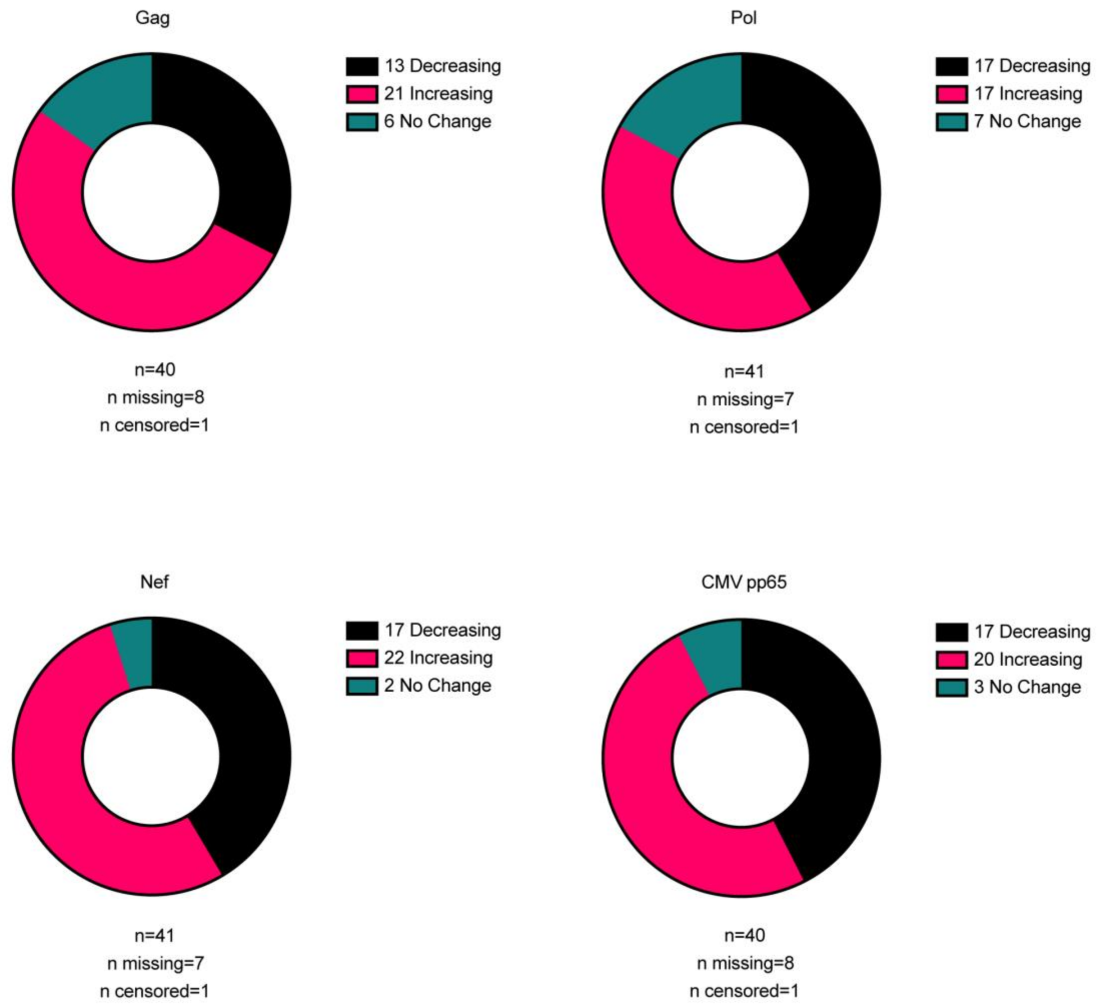

Figure S3. Patterns of longitudinal granzyme B T-cell response changes between 24-168 weeks post-A5321 study entry. Participant T-cell responses were categorized as either increasing, decreasing, or not changing (defined as ≤15% change in either direction) between the two batched on-ART timepoints, and data is shown in parts-of-whole plots. Participants were categorized as missing if they had a missing value for a response at either timepoint. Participants were categorized as censored if they had a censored value for a response at either timepoint.

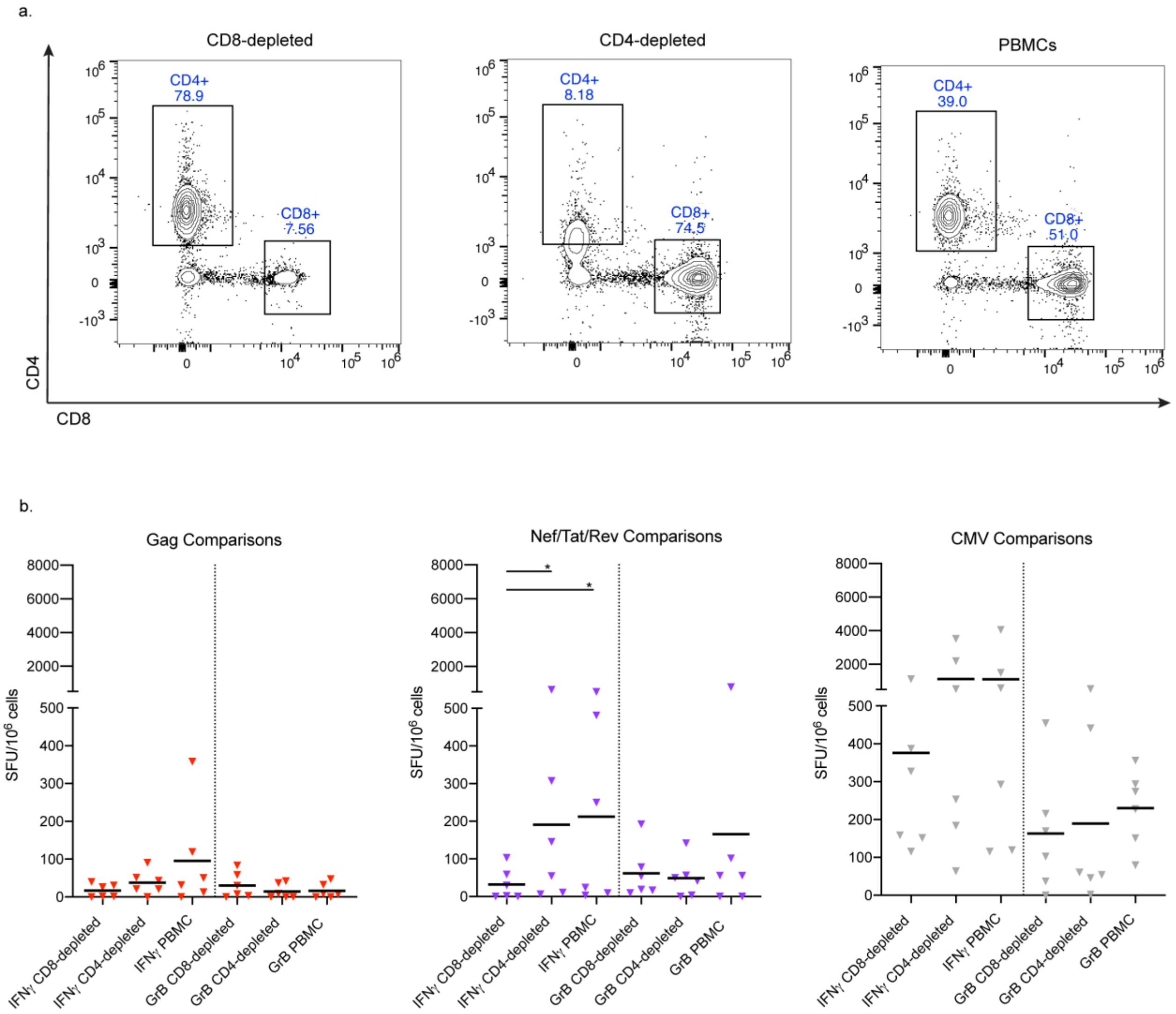

Figure S4. ELISPOT comparisons in cell depletion experiments. A. Representative flow cytometry results from one participant for cell depletion conditions depicting % $CD4^+$ and % $CD8^+$ cells, gated on $CD3^+$ cells from PBMCs. **B.** IFNγ and granzyme B (GrB) ELISPOT results for each cell depletion condition in 6 participants. Cell numbers ranged from 20,000 to 200,000 cells per well. Each data point represents the mean SFU/10^6 cells following background subtraction of negative control wells (duplicates). Horizontal bars represent the mean value for each condition. Conditions were compared by gene product within each ELISPOT assay type using the Friedman test with Dunn's multiple comparisons post hoc test. Asterisks represent p-values adjusted for multiple comparisons (* <0.05, ** <0.01, *** <0.001).

Table S1. Summary statistics for T-cell responses

IFNγ Responses - Study entry

Response	Mean	SD	n	n missing	n censored
Gag	237.6	352.7	49	0	0
Env	48.0	86.1	49	0	0
Pol	253.4	356.4	49	0	0
Nef/Tat/Rev	182.6	262.6	49	0	0
Sum HIV	721.6	761.7	49	0	0
CMV pp65	1319.9	862.1	44	5	0

IFNγ Responses - 24 weeks

Response	Mean	SD	n	n missing	n censored
Gag	297.4	487.9	43	6	0
Env	75.5	118.2	43	6	0
Pol	173.1	241.8	43	6	0
Nef	107.7	130.4	43	6	0
Tat	34.4	56.2	43	6	0
Rev	30.0	52.5	43	6	0
Nef/Tat/Rev	107.5	130.6	43	6	0
Sum HIV	653.6	821.6	43	6	0
CMV pp65	1154.9	1204.0	43	6	0

IFNγ Responses - 168 weeks

Response	Mean	SD	n	n missing	n censored
Gag	169.2	222.6	44	4	1
Env	37.8	67.7	44	4	1
Pol	99.5	144.2	44	4	1
Nef	75.4	113.5	44	4	1
Tat	20.5	35.2	44	4	1
Rev	12.1	20.3	44	4	1
Nef/Tat/Rev	79.0	125.2	44	4	1
Sum HIV	385.6	383.2	44	4	1
CMV pp65	1217.7	1435.0	42	6	1

GrB Responses - 24 weeks

Response	Mean	SD	n	n missing	n censored
Gag	29.3	38.1	45	4	0
Pol	30.7	46.3	46	3	0
Nef	55.5	96.7	46	3	0
CMV pp65	218.1	386.0	45	4	0

GrB Responses - 168 weeks

Response	Mean	SD	n	n missing	n censored
Gag	29.2	45.2	44	4	1
Pol	33.9	42.8	44	4	1
Nef	55.0	85.6	44	4	1
CMV pp65	275.8	407.1	44	4	1

Table S2. Linear mixed-effects model results for differences in HIV-specific T-cell responses between gene products									
IFNγ Responses[A]					*GrB Responses*[A]				
**Comparison**	**Mean Difference**[B]	**95% LCL**	**95% UCL**	**p-Value**[C]	**Comparison**	**Mean Difference**[B]	**95% LCL**	**95% UCL**	**p-Value**[C]
Gag vs. Env	**0.737**	**0.401**	**1.073**	**<0.001**	*Gag vs. Pol*	-0.095	-0.330	0.139	0.602
Gag vs. Pol	0.166	-0.164	0.496	0.698	*Gag vs. Nef*	-0.142	-0.366	0.083	0.297
Gag vs. Nef	**0.597**	**0.275**	**0.918**	**<0.001**	*Pol vs. Nef*	-0.046	-0.270	0.177	0.875
Gag vs. Tat	**0.947**	**0.591**	**1.303**	**<0.001**					
Gag vs. Rev	**1.083**	**0.726**	**1.441**	**<0.001**					
Env vs. Pol	**-0.571**	**-0.912**	**-0.230**	**<0.001**					
Env vs. Nef	-0.140	-0.473	0.193	0.831					
Env vs. Tat	0.210	-0.156	0.577	0.567					
Env vs. Rev	0.346	-0.021	0.714	0.077					
Pol vs. Nef	**0.431**	**0.104**	**0.758**	**0.003**					
Pol vs. Tat	**0.781**	**0.420**	**1.142**	**<0.001**					
Pol vs. Rev	**0.917**	**0.556**	**1.279**	**<0.001**					
Nef vs. Tat	0.351	-0.003	0.704	0.054					
Nef vs. Rev	**0.487**	**0.132**	**0.841**	**0.002**					
Tat vs. Rev	0.136	-0.250	0.522	0.913					

[A]Responses measured at weeks 24 and 168 post-study entry

[B]Least square mean difference in $\log_{10}$ ELISPOT spots/10^6 PBMCs

[C]Adjusted for multiple comparisons using Tukey's method

Note 1: LCL - lower confidence limit; UCL - upper confidence limit

Note 2: significant differences are bolded

Table S3. Linear mixed-effects model results for overall effect of time on T-cell responses					
		IFNγ Responses			
**Response[A]**	**Beta[B]**	**95% LCL**	**95% UCL**	**p-Value**	**Mean % Change per Week (ELISPOT spots/10^6 PBMCs)**
Gag	**-0.00153**	**-0.00277**	**-0.00027**	**0.020**	**-0.35%**
Env	**-0.00271**	**-0.00426**	**-0.00107**	**0.002**	**-0.62%**
Pol	**-0.00239**	**-0.00367**	**-0.00109**	**0.001**	**-0.55%**
Nef	-0.00116	-0.00262	0.00031	0.125	n.s.
Nef/Tat/Rev	-0.00121	-0.00240	-0.00002	0.052	n.s.
Gag + Env + Pol	**-0.00151**	**-0.00265**	**-0.00035**	**0.013**	**-0.35%**
Sum HIV	-0.00099	-0.00212	0.00014	0.090	n.s.
CMV pp65	-0.00048	-0.00141	0.00046	0.319	n.s.
		GrB Responses			
**Response[A]**	**Beta[B]**	**95% LCL**	**95% UCL**	**p-Value**	**Mean % Change per Week (ELISPOT spots/10^6 PBMCs)**
Gag	-0.00077	-0.00249	0.00096	0.386	n.s.
Pol	0.00011	-0.00130	0.00151	0.879	n.s.
Nef	0.00006	-0.00147	0.00157	0.938	n.s.
CMV pp65	**0.00145**	**0.00011**	**0.00282**	**0.040**	**0.33%**

[A]Modeling log_{10}-transformed magnitudes

[B]Modeling effect of time in weeks

Note 1: LCL - lower confidence limit; UCL - upper confidence limit; n.s. - not significant

Note 2: significant effects are bolded

Table S4. Spearman correlations between slopes of change in magnitudes of IFNγ T-cell responses from week 24 to week 168 with virologic and immunologic parameters

Variable		*Gag*	*Env*	*Pol*	*Nef*	*Nef/Tat/Rev*	*Gag + Env + Pol*	*Sum HIV*	*CMV pp65*
	r	0.10	0.13	0.24	**0.51**	0.41	0.26	0.22	0.05
	p-value	0.748	0.592	0.960	**0.012**	0.131	0.331	0.557	0.843
HIV CA-DNA at A5321 entry (cps/10^6 CD4+ T-cells)	n	36	35	37	37	35	39	39	37
	Adjusted^{A} r	0.07	0.16	0.23	**0.50**	0.34	0.25	0.18	-
	Adjusted^{A} p-value	0.927	0.527	0.825	**0.012**	0.450	0.279	0.596	-
	r	0.30	0.22	0.17	**0.46**	0.26	0.31	0.31	0.03
	p-value	0.402	0.408	0.960	**0.018**	0.445	0.291	0.292	0.843
HIV CA-RNA at A5321 entry (cps/10^6 CD4+ T-cells)	n	34	33	35	35	33	37	37	37
	Adjusted^{A} r	0.21	0.19	0.17	0.40	0.20	0.27	0.25	-
	Adjusted^{A} p-value	0.693	0.485	0.825	0.063	0.792	0.279	0.416	-
	r	-0.10	-0.30	-0.02	0.16	-0.01	-0.16	-0.12	0.13
	p-value	0.748	0.315	0.960	0.461	0.989	0.516	0.738	0.583
HIV plasma RNA via iSCA at A5321 entry (cps/mL)	n	35	34	36	36	34	38	38	36
	Adjusted^{A} r	-0.31	-0.42	-0.12	-0.06	-0.14	-0.32	-0.34	-
	Adjusted^{A} p-value	0.347	0.144	0.825	0.729	0.792	0.279	0.192	-
	r	0.03	0.29	0.02	0.01	0.05	0.18	0.06	-0.23
	p-value	0.853	0.315	0.960	0.966	0.989	0.516	0.738	0.309
%PD-1+ CD4+ cells at A5321 entry	n	32	32	33	33	32	35	35	33
	Adjusted^{A} r	<-0.01	0.34	-0.04	-0.07	-0.01	0.15	0.01	-
	Adjusted^{A} p-value	0.997	0.199	0.891	0.729	0.965	0.540	0.966	-
	r	0.08	0.32	0.01	-0.02	<0.01	0.21	0.07	-0.24
	p-value	0.751	0.315	0.960	0.966	0.989	0.495	0.738	0.309
%PD-1+ CD8+ cells at A5321 entry	n	32	32	33	33	32	35	35	33
	Adjusted^{A} r	0.04	0.36	-0.06	-0.11	-0.05	0.18	0.01	-
	Adjusted^{A} p-value	0.966	0.199	0.891	0.729	0.965	0.524	0.966	-
	r	-0.14	-0.14	-0.02	-0.17	0.05	-0.03	-0.11	0.26
	p-value	0.748	0.592	0.960	0.457	0.989	0.855	0.738	0.301
Age at A5321 entry	n	36	35	37	37	35	39	39	37
	Adjusted^{A} r	-0.13	-0.08	0.02	-0.26	-0.01	-0.01	-0.11	-
	Adjusted^{A} p-value	0.926	0.670	0.891	0.283	0.965	0.972	0.821	-
	r	0.16	0.10	-0.11	0.20	0.12	0.09	0.08	0.27
	p-value	0.748	0.642	0.960	0.412	0.864	0.664	0.738	0.301
Years on ART at A5321 entry	n	36	35	37	37	35	39	39	37
	Adjusted^{B} r	0.10	0.08	-0.13	0.18	0.12	0.06	0.05	-
	Adjusted^{B} p-value	0.926	0.670	0.825	0.484	0.792	0.808	0.966	-
	r	0.44	0.23	0.13	**0.47**	0.22	0.30	0.37	0.25
	p-value	0.072	0.408	0.960	**0.014**	0.479	0.291	0.171	0.301
Pre-ART plasma HIV-1 RNA (log_{10}cps/mL)	n	36	35	37	37	35	39	39	37
	Adjusted^{C} r	0.43	0.22	0.11	**0.48**	0.17	0.28	0.36	-
	Adjusted^{C} p-value	0.082	0.438	0.825	**0.012**	0.792	0.279	0.192	-
	r	-0.10	0.08	-0.08	-0.29	-0.26	-0.09	-0.18	-0.43
Pre-ART CD4+ T-cell count (cells/mm^3)	p-value	0.748	0.644	0.960	0.180	0.445	0.664	0.637	0.077
	n	36	35	37	37	35	39	39	37

^{A}Controlling for Pre-ART plasma HIV-1 RNA (log_{10}cps/mL), Pre-ART CD4+ T-cell count (cells/mm^3), and Years on ART at A5321 entry

^{B}Controlling for Pre-ART plasma HIV-1 RNA (log_{10}cps/mL) and Pre-ART CD4+ T-cell count (cells/mm^3)

^{C}Controlling for HIV CA-DNA at A5321 entry (cps/10^6 CD4+ T-cells)

Note 1: zero-value slopes reflecting a change from 0 magnitude to 0 magnitude excluded

Note 2: p-values corrected for false discovery rate

Note 3: significant associations are bolded, and associations which remained significant after controlling for potential confounders are underlined

Table S5. Spearman correlations between slopes of change in magnitudes of IFNγ T-cell responses from week 24 to week 168

		Gag	*Env*	*Pol*	*Nef*	*Nef/Tat/Rev*	*Sum HIV*	*CMV pp65*
Gag	r	-						
	p-value	-						
	n	-						
Env	r	**0.62**	-					
	p-value	**<0.001**	-					
	n	33	-					
Pol	r	**0.57**	**0.36**	-				
	p-value	**0.001**	**0.045**	-				
	n	35	33	-				
Nef	r	**0.56**	**0.44**	**0.45**	-			
	p-value	**0.001**	**0.013**	**0.010**	-			
	n	35	33	36	-			
Nef/Tat/Rev	r	**0.55**	0.36	**0.54**	**0.70**	-		
	p-value	**0.002**	0.054	**0.002**	**<0.001**	-		
	n	33	31	34	34	-		
Sum HIV	r	**0.92**	**0.66**	**0.71**	**0.69**	**0.73**	-	
	p-value	**<0.001**	**<0.001**	**<0.001**	**<0.001**	**<0.001**	-	
	n	36	35	37	37	35	-	
CMV pp65	r	0.32	0.33	0.26	**0.51**	**0.46**	**0.43**	-
	p-value	0.069	0.067	0.127	**0.003**	**0.011**	**0.011**	-
	n	34	33	35	35	33	37	-

Note 1: zero-value slopes reflecting a change from 0 magnitude to 0 magnitude excluded
Note 2: p-values corrected for false discovery rate
Note 3: significant associations are bolded

Table S6. Spearman correlations between slopes of change in log$_{10}$-magnitudes of IFNγ T-cell responses from week 24 to week 168 with virologic and immunologic parameters

Variable		*Gag*	*Env*	*Pol*	*Nef*	*Nef/Tat/Rev*	*Gag + Env + Pol*	*Sum HIV*	*CMV pp65*
HIV CA-DNA at A5321 entry (cps/10^6 CD4+ T-cells)	r	0.20	0.07	0.23	**0.51**	0.38	0.27	0.38	0.01
	p-value	0.928	0.771	0.710	**0.013**	0.202	0.426	0.163	0.956
	n	36	35	37	37	35	39	39	37
	Adjusted^A r	0.22	0.15	0.27	**0.49**	0.36	0.27	0.36	-
	Adjusted^A p-value	0.892	0.598	0.608	**0.029**	0.369	0.532	0.258	-
HIV CA-RNA at A5321 entry (cps/10^6 CD4+ T-cells)	r	0.19	0.05	-0.08	0.33	0.06	0.28	0.34	0.02
	p-value	0.928	0.785	0.710	0.152	0.926	0.426	0.166	0.956
	n	34	33	35	35	33	37	37	37
	Adjusted^A r	0.18	0.06	-0.12	0.27	0.02	0.26	0.31	-
	Adjusted^A p-value	0.892	0.753	0.780	0.368	0.984	0.532	0.289	-
HIV plasma RNA via iSCA at A5321 entry (cps/mL)	r	-0.18	-0.17	0.18	0.23	0.04	-0.12	-0.12	0.12
	p-value	0.928	0.695	0.710	0.321	0.926	0.737	0.609	0.626
	n	35	34	36	36	34	38	38	36
	Adjusted^A r	-0.20	-0.28	0.10	0.11	-0.07	-0.19	-0.25	-
	Adjusted^A p-value	0.892	0.598	0.780	0.860	0.984	0.664	0.394	-
%PD-1+ CD4+ cells at A5321 entry	r	<0.01	0.16	0.09	0.08	0.02	0.17	0.22	-0.19
	p-value	0.984	0.695	0.710	0.862	0.926	0.722	0.384	0.508
	n	32	32	33	33	32	35	35	33
	Adjusted^A r	-0.01	0.15	<-0.01	-0.01	-0.04	0.14	0.16	-
	Adjusted^A p-value	0.980	0.598	0.998	0.973	0.984	0.698	0.515	-
%PD-1+ CD8+ cells at A5321 entry	r	0.09	0.20	0.13	0.05	-0.07	0.21	0.25	-0.17
	p-value	0.984	0.695	0.710	0.871	0.926	0.686	0.384	0.515
	n	32	32	33	33	32	35	35	33
	Adjusted^A r	0.07	0.17	0.03	-0.04	-0.12	0.18	0.19	-
	Adjusted^A p-value	0.980	0.598	0.998	0.973	0.984	0.664	0.466	-
Age at A5321 entry	r	<-0.01	-0.08	0.13	0.01	0.06	-0.01	0.02	0.40
	p-value	0.984	0.771	0.710	0.968	0.926	0.953	0.909	0.137
	n	36	35	37	37	35	39	39	37
	Adjusted^A r	<0.01	0.07	0.25	-0.03	0.08	<0.01	0.03	-
	Adjusted^A p-value	0.980	0.753	0.608	0.973	0.984	0.984	0.932	-
Years on ART at A5321 entry	r	0.07	-0.13	-0.08	0.16	0.02	0.03	0.03	0.24
	p-value	0.984	0.695	0.710	0.533	0.926	0.953	0.909	0.464
	n	36	35	37	37	35	39	39	37
	Adjusted^B r	0.06	-0.17	-0.13	0.14	<0.01	0.01	0.01	-
	Adjusted^B p-value	0.980	0.598	0.780	0.860	0.984	0.984	0.932	-
Pre-ART plasma HIV-1 RNA (log$_{10}$cps/mL)	r	0.06	0.15	0.22	0.33	0.21	0.13	0.21	0.21
	p-value	0.984	0.695	0.710	0.152	0.926	0.737	0.384	0.484
	n	36	35	37	37	35	39	39	37
	Adjusted^C r	0.04	0.14	0.21	0.31	0.16	0.10	0.19	-
	Adjusted^C p-value	0.980	0.598	0.608	0.266	0.984	0.728	0.466	-
Pre-ART CD4+ T-cell count (cells/mm^3)	r	0.03	0.22	0.03	-0.23	-0.14	-0.04	-0.14	-0.32
	p-value	0.984	0.695	0.847	0.321	0.926	0.953	0.601	0.248
	n	36	35	37	37	35	39	39	37

^{A}Controlling for Pre-ART plasma HIV-1 RNA (log$_{10}$cps/mL), Pre-ART CD4+ T-cell count (cells/mm^3), and Years on ART at A5321 entry

^{B}Controlling for Pre-ART plasma HIV-1 RNA (log$_{10}$cps/mL) and Pre-ART CD4+ T-cell count (cells/mm^3)

^{C}Controlling for HIV CA-DNA at A5321 entry (cps/10^6 CD4+ T-cells)

Note 1: zero-value slopes reflecting a change from 0 magnitude to 0 magnitude excluded

Note 2: slopes reflecting a change from 0 magnitude to a non-zero magnitude set to highest rank

Note 3: slopes reflecting a change from a non-zero magnitude to 0 magnitude set to lowest rank

Note 4: p-values corrected for false discovery rate

Note 5: significant associations are bolded, and associations which remained significant after controlling for potential confounders are underlined

Table S7. Spearman correlations between slopes of change in log_{10}-magnitudes of IFNγ T-cell responses from week 24 to week 168

		Gag	*Env*	*Pol*	*Nef*	*Nef/Tat/Rev*	*Sum HIV*	*CMV pp65*
Gag	r	-						
	p-value	-						
	n	-						
Env	r	**0.47**	-					
	p-value	**0.012**	-					
	n	33	-					
Pol	r	**0.54**	**0.43**	-				
	p-value	**0.003**	**0.024**	-				
	n	35	33	-				
Nef	r	**0.49**	0.30	**0.41**	-			
	p-value	**0.007**	0.112	**0.023**	-			
	n	35	33	36	-			
Nef/Tat/Rev	r	**0.39**	**0.42**	**0.54**	**0.59**	-		
	p-value	**0.037**	**0.030**	**0.003**	**0.001**	-		
	n	33	31	34	34	-		
Sum HIV	r	**0.90**	**0.66**	**0.64**	**0.61**	**0.60**	-	
	p-value	**<0.001**	**<0.001**	**<0.001**	**<0.001**	**<0.001**	-	
	n	36	35	37	37	35	-	
CMV pp65	r	0.10	0.28	0.08	0.24	**0.39**	0.16	-
	p-value	0.583	0.146	0.633	0.199	**0.037**	0.379	-
	n	34	33	35	35	33	37	-

Note 1: zero-value slopes reflecting a change from 0 magnitude to 0 magnitude excluded
Note 2: slopes reflecting a change from 0 magnitude to a non-zero magnitude set to highest rank
Note 3: slopes reflecting a change from a non-zero magnitude to 0 magnitude set to lowest rank
Note 4: p-values corrected for false discovery rate
Note 5: significant associations are bolded

**Table S8. Linear mixed-effects model results for overall effect of time from week 24 to week 168 on Nef-specific IFNγ T-cell responses using >3x background positivity cutoff**					
**Response[A]**	**Beta[B]**	**95% LCL**	**95% UCL**	**p-Value**	**Mean % Change per Week (ELISPOT spots/10^6 PBMCs)**
Nef	-0.00117	-0.00263	0.00034	0.127	n.s.

[A]Modeling log_{10}-transformed magnitudes

[B]Modeling effect of time in weeks

Note 1: LCL - lower confidence limit; UCL - upper confidence limit; n.s. - not significant

Table S9. Spearman correlations between slopes of change in magnitudes of Nef-specific IFNγ T-cell responses from week 24 to week 168 with virologic and immunologic parameters using >3x background positivity cutoff

**Variable**		*Nef*
HIV CA-DNA at A5321 entry (cps/10^6 CD4+ T-cells)	r	**0.66**
	p-value	**<0.001**
	n	29
	Adjusted[A] r	**0.63**
	Adjusted[A] p-value	**0.001**
HIV CA-RNA at A5321 entry (cps/10^6 CD4+ T-cells)	r	**0.50**
	p-value	**0.008**
	n	27
	Adjusted[A] r	**0.43**
	Adjusted[A] p-value	**0.035**
HIV plasma RNA via iSCA at A5321 entry (cps/mL)	r	0.16
	p-value	0.425
	n	28
	Adjusted[A] r	0.03
	Adjusted[A] p-value	0.875
%PD-1+ CD4+ cells at A5321 entry	r	-0.02
	p-value	0.913
	n	26
	Adjusted[A] r	-0.07
	Adjusted[A] p-value	0.738
%PD-1+ CD8+ cells at A5321 entry	r	-0.09
	p-value	0.666
	n	26
	Adjusted[A] r	-0.13
	Adjusted[A] p-value	0.555
Age at A5321 entry	r	-0.17
	p-value	0.368
	n	29
	Adjusted[A] r	-0.24
	Adjusted[A] p-value	0.229
Years on ART at A5321 entry	r	0.18
	p-value	0.354
	n	29
	Adjusted[B] r	0.14
	Adjusted[B] p-value	0.501
Pre-ART plasma HIV-1 RNA (log_{10}cps/mL)	r	0.34
	p-value	0.070
	n	29
	Adjusted[C] r	0.23
	Adjusted[C] p-value	0.239
Pre-ART CD4+ T-cell count (cells/mm^3)	r	-0.20
	p-value	0.303
	n	29

[A]Controlling for Pre-ART plasma HIV-1 RNA (log_{10}cps/mL), Pre-ART CD4+ T-cell count (cells/mm^3), and Years on ART at A5321 entry

[B]Controlling for Pre-ART plasma HIV-1 RNA (log_{10}cps/mL) and Pre-ART CD4+ T-cell count (cells/mm^3)

[C]Controlling for HIV CA-DNA at A5321 entry (cps/10^6 CD4+ T-cells)

Note 1: zero-value slopes reflecting a change from 0 magnitude to 0 magnitude excluded

Note 2: slopes reflecting a change from 0 magnitude to a non-zero magnitude set to highest rank

Note 3: slopes reflecting a change from a non-zero magnitude to 0 magnitude set to lowest rank

**Table S10. Spearman correlations between slopes of change in magnitudes of GrB T-cell responses from week 24 to week 168 with virologic and immunologic parameters**						
**Variable**		*Gag*	*Pol*	*Nef*	*Gag + Pol*	*CMV pp65*
HIV CA-DNA at A5321 entry (cps/10^6 CD4+ T-cells)	r	-0.20	-0.27	-0.14	-0.18	0.09
	p-value	0.569	0.920	0.954	0.627	0.964
	n	36	37	41	39	40
	Adjusted[A] r	-0.17	-0.25	-0.10	-0.13	-
	Adjusted[A] p-value	0.464	0.928	0.967	0.583	-
HIV CA-RNA at A5321 entry (cps/10^6 CD4+ T-cells)	r	0.17	0.07	-0.08	0.16	0.41
	p-value	0.573	0.986	0.954	0.627	0.079
	n	35	36	40	38	39
	Adjusted[A] r	0.18	0.08	-0.10	0.16	-
	Adjusted[A] p-value	0.464	0.928	0.967	0.583	-
HIV plasma RNA via iSCA at A5321 entry (cps/mL)	r	-0.32	-0.08	0.02	-0.23	-0.05
	p-value	0.491	0.986	0.954	0.627	0.964
	n	34	35	39	37	38
	Adjusted[A] r	-0.30	0.02	0.08	-0.19	-
	Adjusted[A] p-value	0.418	0.928	0.967	0.583	-
%PD-1+ CD4+ cells at A5321 entry	r	0.22	-0.05	-0.11	0.11	0.04
	p-value	0.569	0.986	0.954	0.726	0.964
	n	32	32	36	35	35
	Adjusted[A] r	0.25	-0.03	-0.09	0.14	-
	Adjusted[A] p-value	0.464	0.928	0.967	0.583	-
%PD-1+ CD8+ cells at A5321 entry	r	0.29	-0.01	-0.01	0.17	0.01
	p-value	0.491	0.986	0.954	0.627	0.964
	n	32	32	36	35	35
	Adjusted[A] r	0.32	0.02	0.01	0.20	-
	Adjusted[A] p-value	0.418	0.928	0.967	0.583	-
Age at A5321 entry	r	-0.13	0.01	-0.01	-0.06	-0.18
	p-value	0.573	0.986	0.954	0.728	0.770
	n	36	37	41	39	40
	Adjusted[A] r	-0.20	-0.08	-0.02	-0.11	-
	Adjusted[A] p-value	0.464	0.928	0.967	0.583	-
Years on ART at A5321 entry	r	0.13	0.15	0.12	0.15	0.26
	p-value	0.573	0.986	0.954	0.627	0.449
	n	36	37	41	39	40
	Adjusted[B] r	0.13	0.14	0.13	0.16	-
	Adjusted[B] p-value	0.528	0.928	0.967	0.583	-
Pre-ART plasma HIV-1 RNA (log_{10}cps/mL)	r	-0.09	-0.14	-0.03	-0.08	-0.01
	p-value	0.695	0.986	0.954	0.726	0.964
	n	36	37	41	39	40
	Adjusted[C] r	-0.03	-0.07	0.01	-0.04	-
	Adjusted[C] p-value	0.885	0.928	0.967	0.816	-
Pre-ART CD4+ T-cell count (cells/mm^3)	r	0.04	<0.01	0.18	0.08	0.04
	p-value	0.813	0.986	0.954	0.726	0.964
	n	36	37	41	39	40

[A]Controlling for Pre-ART plasma HIV-1 RNA (log_{10}cps/mL), Pre-ART CD4+ T-cell count (cells/mm^3), and Years on ART at A5321 entry

[B]Controlling for Pre-ART plasma HIV-1 RNA (log_{10}cps/mL) and Pre-ART CD4+ T-cell count (cells/mm^3)

[C]Controlling for HIV CA-DNA at A5321 entry (cps/10^6 CD4+ T-cells)

Note 1: zero-value slopes reflecting a change from 0 magnitude to 0 magnitude excluded

Note 2: p-values corrected for false discovery rate

Table S11. Spearman correlations between slopes of change in magnitudes of GrB T-cell responses from week 24 to week 168

		Gag	*Pol*	*Nef*	*CMV pp65*
Gag	r	-			
	p-value	-			
	n	-			
Pol	r	**0.59**	-		
	p-value	**<0.001**	-		
	n	33	-		
Nef	r	**0.36**	**0.41**	-	
	p-value	**0.031**	**0.011**	-	
	n	36	37	-	
CMV pp65	r	**0.38**	**0.42**	**0.54**	-
	p-value	**0.022**	**0.011**	**<0.001**	-
	n	36	36	40	-

Note 1: zero-value slopes reflecting a change from 0 magnitude to 0 magnitude excluded

Note 2: p-values corrected for false discovery rate

Note 3: significant associations are bolded

Table S12. Spearman correlations between slopes of change in log_{10}-magnitudes of GrB T-cell responses from week 24 to week 168 with virologic and immunologic parameters

Variable		*Gag*	*Pol*	*Nef*	*Gag + Pol*	*CMV pp65*
HIV CA-DNA at A5321 entry (cps/10^6 CD4+ T-cells)	r	0.12	-0.23	-0.12	-0.09	0.02
	p-value	0.839	0.999	0.913	0.751	0.922
	n	36	37	41	39	40
	Adjusted[A] r	0.14	-0.23	-0.08	-0.04	-
	Adjusted[A] p-value	0.689	0.957	0.988	0.973	-
HIV CA-RNA at A5321 entry (cps/10^6 CD4+ T-cells)	r	0.27	-0.03	-0.07	0.17	0.30
	p-value	0.344	0.999	0.913	0.710	0.301
	n	35	36	40	38	39
	Adjusted[A] r	0.26	-0.05	-0.09	0.15	-
	Adjusted[A] p-value	0.320	0.995	0.988	0.776	-
HIV plasma RNA via iSCA at A5321 entry (cps/mL)	r	-0.40	-0.09	0.02	-0.17	-0.12
	p-value	0.158	0.999	0.913	0.710	0.922
	n	34	35	39	37	38
	Adjusted[A] r	-0.46	-0.02	0.06	-0.12	-
	Adjusted[A] p-value	0.077	0.995	0.988	0.797	-
%PD-1+ CD4+ cells at A5321 entry	r	0.25	-0.06	-0.09	0.16	0.06
	p-value	0.371	0.999	0.913	0.710	0.922
	n	32	32	36	35	35
	Adjusted[A] r	0.27	-0.02	-0.08	0.20	-
	Adjusted[A] p-value	0.320	0.995	0.988	0.776	-
%PD-1+ CD8+ cells at A5321 entry	r	0.31	-0.05	-0.02	0.15	0.08
	p-value	0.344	0.999	0.913	0.710	0.922
	n	32	32	36	35	35
	Adjusted[A] r	0.33	<0.01	-0.01	0.19	-
	Adjusted[A] p-value	0.320	0.995	0.988	0.776	-
Age at A5321 entry	r	-0.04	0.09	0.02	0.02	-0.11
	p-value	0.839	0.999	0.913	0.908	0.922
	n	36	37	41	39	40
	Adjusted[A] r	-0.03	0.02	0.06	-0.03	-
	Adjusted[A] p-value	0.937	0.995	0.988	0.973	-
Years on ART at A5321 entry	r	0.06	0.20	0.02	0.22	0.35
	p-value	0.839	0.999	0.913	0.710	0.247
	n	36	37	41	39	40
	Adjusted[B] r	0.07	0.20	0.03	0.22	-
	Adjusted[B] p-value	0.937	0.957	0.988	0.776	-
Pre-ART plasma HIV-1 RNA (log_{10}cps/mL)	r	0.05	-0.01	-0.03	-0.02	0.02
	p-value	0.839	0.999	0.913	0.908	0.922
	n	36	37	41	39	40
	Adjusted[C] r	0.01	0.05	<0.01	0.01	-
	Adjusted[C] p-value	0.937	0.995	0.988	0.976	-
Pre-ART CD4+ T-cell count (cells/mm^3)	r	0.08	<-0.01	0.19	0.09	0.05
	p-value	0.839	0.999	0.913	0.751	0.922
	n	36	37	41	39	40

[A]Controlling for Pre-ART plasma HIV-1 RNA (log_{10}cps/mL), Pre-ART CD4+ T-cell count (cells/mm^3), and Years on ART at A5321 entry

[B]Controlling for Pre-ART plasma HIV-1 RNA (log_{10}cps/mL) and Pre-ART CD4+ T-cell count (cells/mm^3)

[C]Controlling for HIV CA-DNA at A5321 entry (cps/10^6 CD4+ T-cells)

Note 1: zero-value slopes reflecting a change from 0 magnitude to 0 magnitude excluded

Note 2: slopes reflecting a change from 0 magnitude to a non-zero magnitude set to highest rank

Note 3: slopes reflecting a change from a non-zero magnitude to 0 magnitude set to lowest rank

Note 4: p-values corrected for false discovery rate

Table S13. Spearman correlations between slopes of change in log_{10}-magnitudes of GrB T-cell responses from week 24 to week 168

		Gag	*Pol*	*Nef*	*CMV pp65*
Gag	r	-			
	p-value	-			
	n	-			
Pol	r	0.26	-		
	p-value	0.137	-		
	n	33	-		
Nef	r	**0.43**	**0.55**	-	
	p-value	**0.011**	**0.001**	-	
	n	36	37	-	
CMV pp65	r	**0.58**	**0.54**	**0.63**	-
	p-value	**0.001**	**0.001**	**0.001**	-
	n	36	36	40	-

Note 1: zero-value slopes reflecting a change from 0 magnitude to 0 magnitude excluded
Note 2: slopes reflecting a change from 0 magnitude to a non-zero magnitude set to highest rank
Note 3: slopes reflecting a change from a non-zero magnitude to 0 magnitude set to lowest rank
Note 4: p-values corrected for false discovery rate
Note 5: significant associations are bolded

Supplemental Acknowledgments

We would like to acknowledge all the members of the A5321 team, including Evelyn Hogg, Rebecca LeBlanc, Christine Scello, David Palm, Monica Gandhi, Courtney Fletcher, Catherine Godfrey, Anthony Podany, Elias Halvas, Joan Dragavon, Jeymohan Joseph, Rose Lagattuta, Leyi Lin, Hannah Mar, Susan Pederson, Kevin Robertson, Leah Rubin, Serena Spudich, Bernadette Jarocki, Sean Avedissian, Ann Collier, Jonathan Li, Sharon Riddler, Aimee Willett, Charles Rinaldo Jr., and Jenny Nguyen.

Below is a listing of all of the above individuals and their affiliations.

Name	Affiliation
Evelyn Hogg	ACTG Network Coordinating Center Social & Scientific Systems<br>8757 Georgia Avenue, 12th Floor Silver Spring, MD 20910-3714
Rebecca LeBlanc	Frontier Science & Technology Research Foundation, Inc.<br>4033 Maple Road<br>Amherst, NY 14226

Christine Scello	Frontier Science & Technology Research Foundation, Inc.<br>Data Management Center<br>4033 Maple Road<br>Amherst, NY 14226-1056
David Palm	Chapel Hill CRS<br><br>P.O. Box 12161<br>Research Triangle Park, NC 27709
Monica Gandhi	University of California San Francisco Division of HIV/AIDS, Box 1352<br>405 Irving Street, 2nd Floor<br>San Francisco, CA 94122-1352
Courtney V. Fletcher	College of Pharmacy<br>University of Nebraska Medical Center 986000 Nebraska Medical Center Omaha, NE 68198-6000
Catherine Godfrey	HIV Research Branch TRP/DAIDS/NIAID/NIH<br><br>5601 Fishers Lane Room 9E49 MSC 9830<br>Bethesda, MD 20852-9830
Anthony Podany	Antiviral Pharmacology Laboratory College of Pharmacy, RM 4007 University of Nebraska Medical Center 986045 Nebraska Medical Center Omaha, NE 68198-6045
Elias Halvas	University of Pittsburgh Virology Support Laboratory<br>Division of Infectious Diseases S813 Scaife Hall<br>3550 Terrace Street<br>Pittsburgh, PA 15261
Joan Dragavon	Virology Specialty Laboratory University of Washington<br>Research & Training Building<br>300 9th Avenue, Room 725<br>Seattle WA 98104-2499

Jeymohan Joseph	HIV Neuropathogenesis and Treatment Branch<br>National Institute of Mental Health Room 6219, MSC 9619<br>6001 Executive Boulevard<br>Bethesda, MD 20892-9619
Rose Lagattuta	11075 Santa Monica Boulevard, Ste. 200 Los Angeles, CA 90025
Leyi Lin	5601 Fishers Lane<br><br>MSC 9830, Room 9E47 Rockville, MD 20852
Hanna Mar	Statistical and Data Analysis Center Harvard School of Public Health FXB 643A<br>Boston, MA 02115
Susan Pedersen	University of North Carolina at Chapel Hill Bioinformatics Building, Suite 2100<br>130 Mason Farm Road<br>Chapel Hill, NC 27599-7215
Kevin Robertson	Chapel Hill CRS<br>Department of Neurology<br>2128 Physician Office Building 170 Manning Drive<br><br>Chapel Hill, NC 27599-7025
Leah H. Rubin	Department of Neurology Johns Hopkins University 600 N. Wolfe Street<br>Meyer 6-113a<br>Baltimore MD 21287
Serena S. Spudich	Cornell CRS<br>Yale University<br>PO Box 208018

	15 York Street<br>New Haven, CT 06520
Bernadette Jarocki	Frontier Science & Technology Research Foundation, Inc.<br>4033 Maple Road<br>Amherst, NY 14226-1056
Sean Avedissian	University of Nebraska Medical Center 42nd and Emile<br>Omaha NE 68198
Ann Collier	University of Washington School of Medicine<br>Harborview Medical Center<br>Box 359929<br>325 9th Avenue<br>Seattle, WA 98104
Jonathan Li	Brigham and Women's Hospital 65 Landsdowne Street, Rm 421 Cambridge, MA 02139
Sharon Riddler	University of Pittsburgh CRS 3520 Fifth Avenue<br>Keystone Building, Suite 510 Pittsburgh, PA 15213-2582
Aimee Willett	Frontier Science & Technology Research Foundation, Inc. 4033 Maple Road Amherst, NY 4226-1056
Charles Rinaldo Jr.	Infectious Diseases & Microbiology University of Pittsburgh A419 Crabtree Hall 130 DeSoto Street Pittsburgh, PA 15261<br>Phone: 412-624-3928
Jenny Nguyen	Laboratory Science Group ACTG Network Coordinating Center Social & Scientific Systems, Inc.8757 Georgia Avenue, 12th Floor Silver Spring, MD 20910
